# Rich-club connectivity, diverse population coupling, and dynamical activity patterns emerging from local cortical circuits

**DOI:** 10.1371/journal.pcbi.1006902

**Published:** 2019-04-02

**Authors:** Yifan Gu, Yang Qi, Pulin Gong

**Affiliations:** 1 School of Physics, University of Sydney, New South Wales, Australia; 2 ARC Centre of Excellence for Integrative Brain Function, University of Sydney, New South Wales, Australia; Duke University, UNITED STATES

## Abstract

Experimental studies have begun revealing essential properties of the structural connectivity and the spatiotemporal activity dynamics of cortical circuits. To integrate these properties from anatomy and physiology, and to elucidate the links between them, we develop a novel cortical circuit model that captures a range of realistic features of synaptic connectivity. We show that the model accounts for the emergence of higher-order connectivity structures, including highly connected hub neurons that form an interconnected rich-club. The circuit model exhibits a rich repertoire of dynamical activity states, ranging from asynchronous to localized and global propagating wave states. We find that around the transition between asynchronous and localized propagating wave states, our model quantitatively reproduces a variety of major empirical findings regarding neural spatiotemporal dynamics, which otherwise remain disjointed in existing studies. These dynamics include diverse coupling (correlation) between spiking activity of individual neurons and the population, dynamical wave patterns with variable speeds and precise temporal structures of neural spikes. We further illustrate how these neural dynamics are related to the connectivity properties by analysing structural contributions to variable spiking dynamics and by showing that the rich-club structure is related to the diverse population coupling. These findings establish an integrated account of structural connectivity and activity dynamics of local cortical circuits, and provide new insights into understanding their working mechanisms.

## Author summary

To integrate essential anatomical and physiological properties of local cortical circuits and to elucidate mechanistic links between them, we develop a novel circuit model capturing key synaptic connectivity features. We show that the model explains the emergence of a range of connectivity patterns such as rich-club connectivity, and gives rise to a rich repertoire of cortical states. We identify both the anatomical and physiological mechanisms underlying the transition of these cortical states, and show that our model reconciles an otherwise disparate set of key physiological findings on neural activity dynamics. We further illustrate how these neural dynamics are related to the connectivity properties by analysing structural contributions to variable spiking dynamics and by showing that the rich-club structure is related to diverse neural population correlations as observed recently. Our model thus provides a framework for integrating and explaining a variety of neural connectivity properties and spatiotemporal activity dynamics observed in experimental studies, and provides novel experimentally testable predictions.

## Introduction

An essential step toward understanding cortical circuits is to interrelate their connectivity and their spatiotemporal dynamics that underlie brain functions [1, 2]. A growing body of work has begun uncovering the basic connectivity properties of cortical circuits at the synaptic level, including distance-dependent connectivity, i.e. the connection probability between neuron pairs decreases as their distance increases [3, 4], and the common-neighbor property, i.e. the connection probability between neuron pairs increases with the number of their shared neighbors [3]. Local cortical circuits with these connectivity properties also possess significant heterogeneity in synaptic efficacy [5, 6], as well as in the number of connections each neuron sends (out-degree) and receives (in-degree), as strongly suggested by both transfer entropy-based effective connectivity measures [7, 8] and anatomically constrained modeling studies [9, 10].

Cortical circuits with such connectivity properties exhibit complex spatial and temporal dynamics. It has been experimentally established that cortical neurons *in vivo* fire very variably [11]. Recent recordings in the cortex of awake mice and monkeys have also revealed that cortical neurons differ in their coupling to the overall firing of the population, ranging from strongly correlated “choristers” to weakly correlated “soloists” [12]. Despite these variable and diverse neural response properties, it has been found that at the level of neural circuits, there exist structured spatiotemporal patterns, such as propagating waves [13–15] and precisely timed spiking triplets [14]. However, it remains unclear whether and how these seemingly distinct neural dynamics at different neural levels can be reconciled, and how these neural dynamics relate to the underlying network structure.

Here, we first develop a generative connectivity model of local cortical circuits, which captures the key connectivity properties found at the synaptic level (i.e., the distance-dependent connectivity, the common-neighbor property, and the heterogeneous degrees). The model predicts and explains the emergence of high-order connectivity patterns, such as overrepresented three-neuron motifs and rich-club connectivity. The former has been observed in rat visual cortex [5], and the latter has been recently observed in a biologically constrained *in silico* model [10] and in the effective connectivity quantified by information transfer between neurons in rodent somatosensory cortex [16]; the existence of high firing rate neurons that tend to be more connected to each other implies such a heterogeneous connectivity [17]. We then construct a biophysically-based, local cortical circuit model of spiking neurons by incorporating the connectivity structure arising from the generative model and by considering an essential neurophysiological property, i.e. correlated excitatory and inhibitory inputs into individual neurons with an equalized I-E ratio across neurons [18]. We find that the cortical circuit exhibits a rich repertoire of collective dynamics ranging from irregular, asynchronous behavior to localized and global propagating waves, modulated by both anatomical and physiological mechanisms.

We illustrate that around this transition point, the circuit model can quantitatively reproduce, as well as provide insights into, a wide range of seemingly contrasting dynamics at different levels, which otherwise remain disjointed in existing modeling studies of cortical circuits [9, 19–22]. These dynamics include tightly balanced excitatory and inhibitory currents into individual neurons [23], synaptic conductance dynamics [24], variability of spiking dynamics [11], diverse coupling relationships between individual neurons and the population [12], dynamical wave patterns occurring intermittently across cortical space with their dynamical properties quantitatively comparable to those measured in [14] and [13], and precise spiking triplet structures [14]. We further characterize how these dynamics relate to the structure by analyzing the contribution of the variability of the heterogeneous connectivity to that of neural activity and by showing how the common-neighbor property provides a novel connectivity mechanism for altering the circuit activity state. Moreover, we demonstrate that the diverse population coupling behavior emerging near the transition point is related to the rich-club connectivity. Our model provides a framework for integrating and explaining a great variety of neural connectivity properties and spatiotemporal activity dynamics observed in experimental studies, thus significantly advancing our understanding of local cortical circuits.

## Results

We develop the local cortical circuit model in two steps. First, we develop a generative connectivity model to capture a wide range of neural connectivity features found at the synaptic level (see Materials and methods). Second, based on the structure generated by the connectivity model, we incorporate experimentally established neurophysiological properties, such as equalized inhibition-excitation (I-E) ratios across neurons [18], to construct a biophysically-based, local cortical circuit model of spiking neurons (see Materials and methods).

### A generative connectivity model of local cortical circuits

We obtain the connectivity structure of our network by iterating the generative model until it converges (see Materials and methods); this structure integrates a range of key connectivity properties of local cortical circuits, including the heterogeneous in- and out-degree distributions [8–10], the distance dependent connectivity [3, 4], the common neighbor property [3]. The generated connectivity structure exhibits emergent, higher-order connectivity patterns, such as overrepresented three-neuron (triad) motifs and rich-club structure.

**Fig 1:**
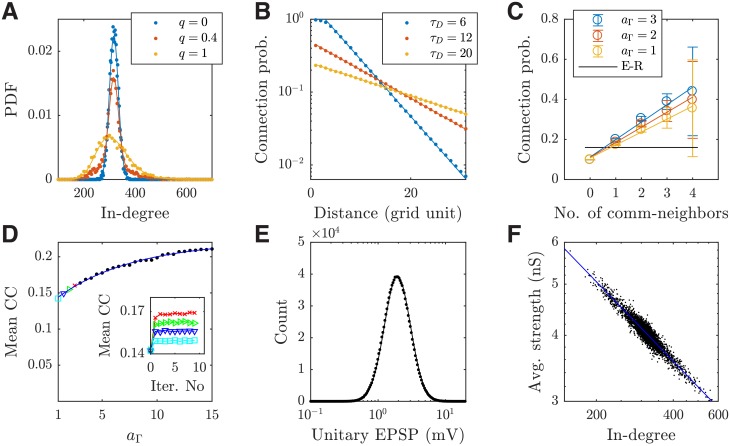
Connectivity properties synthesized by the generative model. (***A***) The generated (dots) and theoretical (solid lines) in-degree distributions with different hybrid parameters *q*; *q* = 0 and *q* = 1 correspond to the pure Poisson and log-normal distributions, respectively. (***B***) The generated (dots) connection probabilities as a function of inter-neuron distance with different decay constants *τ*_*D*_. The decay constants of fitted exponential curves (solid lines) are 5.57 ± 0.05, 11.17 ± 0.04, 19.25 ± 0.15 (95% confidence), respectively. (***C***) The generated (dots) connection probabilities as a function of shared pre-synaptic common neighbors with different common neighbor coefficients *a*_Γ_; the error bars show one SEM of 65 trials. The solid colored lines are the linear fit to the data. For each trial, the results are calculated from 2000 samples; each sample consists of 12 neurons randomly selected from a circular region in the network containing 2000 excitatory neurons to approximate the sampling methods used in [3]. The results are not sensitive to the diameter of the sampling area. The solid black line is the expected curve from the E-R random networks. (***D***) The mean clustering coefficient (CC) of the network (colored and black dots) increases as the common neighbor coefficient *a*_Γ_ increases. The solid line is the fitted exponential function (0.22 − 0.089*e*^−*a*_Γ_/7.04^; *R*^2^ = 0.996). The convergence of mean CC for 4 example *a*_Γ_ values (colored dots) after iterations of the algorithm is shown in the inset. (***E***) The generated (dots) and theoretical (solid line; log-normal) distributions of unitary EPSP magnitudes. (***F***) The average incoming connection strength and the in-degree of individual excitatory neurons (dots) follow the inverse square root scaling (solid line fitted; *R*^2^ = 0.917); note the plot is on a log-log scale. All the empirical data sets or points in (A-B) and (D-F) show the results of individual realizations of the generative model with default parameter values (unless otherwise stated).

As shown in Fig 1*A*, the model accurately generates the given degree distribution specified in Eq 3, with the variance of the default distribution (at *q* = 0.4) over three times larger than that of the Poisson distribution (*q* = 0). The connection probability decreases as the inter-neuron distance increases in the generated networks, as described by the exponential function in our model (Fig 1*B*; see Materials and methods); this is consistent with experimental results in [3] and [4]. The distance decay constants in Fig 1*B* network results are primarily determined by the decay constant *τ*_*D*_ used in our generative model, but also modulated by the common neighbor factor. To illustrate this, we remove the common neighbor factor in the model (by setting *a*_Γ_ = 1) and find that the generated decay constants (6.08 ± 0.04, 12.07 ± 0.07, 20.15 ± 0.12; fitted to one random trial, 95% confidence interval) become almost identical with the generative decay constants *τ*_*D*_ used in our model (*τ*_*D*_ = 6, 12, 20, respectively). As shown in [25], such distance-dependent connections alone can give rise to the common neighbor property in our generated networks; that is, the connection probability increases linearly with the number of shared pre-synaptic common neighbors (Fig 1*C*; *a*_Γ_ = 1; *r* = 0.64, *p* < 0.001). This common neighbor property has been found in layer 5 of rat somatosensory cortex [3].

The additional incorporation of the explicit common neighbor factor in our model (Eq 1), however, allows for a direct control of the slope of increase as shown in Fig 1*C* (*r* = 0.77, *p* < 0.001, for the default model with *a*_Γ_ = 2); this indicates that a stronger common effect (i.e., a greater *a*_Γ_) results in a greater degree of clustering in the generated network (Fig 1*D*), which is a key anatomical mechanism underlying the transition of different activity states in our circuit model as illustrated in the following sections. For the connection strengths in the generated network, their distribution follows the theoretical one (Fig 1*E*), which is log-normal as found in experimental studies [5], and the average incoming connection strengths and in-degrees follow the inverse square root scaling (Fig 1*F*), consistent with [26].

#### Overrepresented connectivity patterns

**Fig 2:**
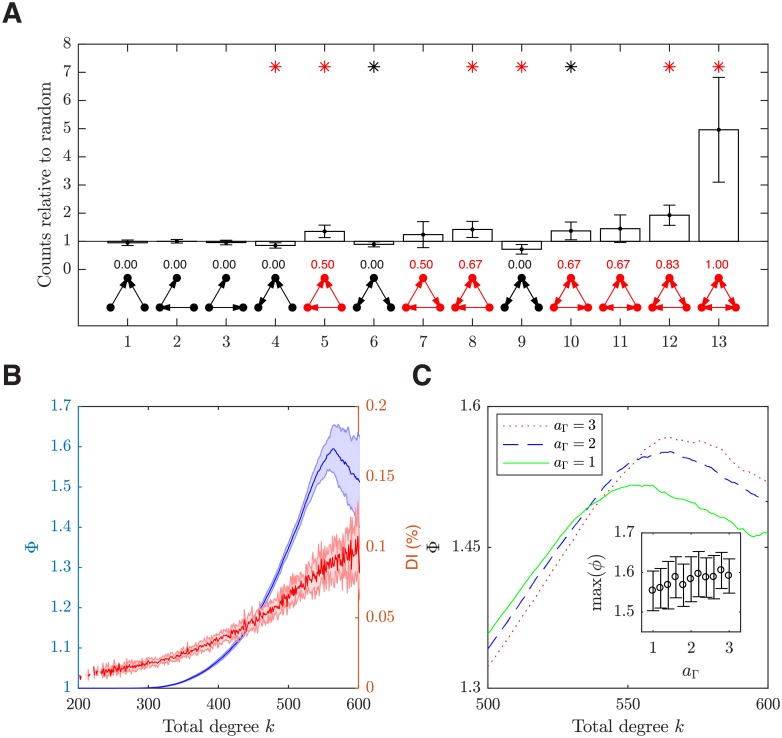
Emergent higher-order connectivity features. (***A***) Triad motif counts. Within each trial, the motif counts are averaged from 1000 randomly sampled quadruplets using the brain connectivity toolbox [28] and 100 control networks are generated to normalize the motif counts. The error bars show one SEM from 10 trials. The mean clustering coefficient is shown above each motif, with the non-zero ones highlighted in red. The asterisks denote the relative motif counts that are statistically significantly different from one (red asterisks *p* < 0.001, black asterisks *p* < 0.034; Holm step-down adjusted p-values [29]). (***B***) Left y-axis (blue): normalized rich-club coefficients Φ(*k*), which measures the extent to which the neurons with total degrees > *k* in a network are over-connected to each other. Within each trial, 100 control networks are generated to normalize the coefficients. The coefficient peaks at 1.60, which is significantly higher than 1 as given by the control networks (*p* < 0.001, one-sample t-test). Right y-axis (red): dynamical importance (averaged across neurons with the same total degree *k* within each trial), defined as the fractional change (%) in the largest eigenvalue of the adjacency matrix of the network upon removal of the neuron from it. The solid lines show the averages over 10 trials and the shaded areas show one SEM. Data for *k* < 200 is omitted from the plot because neurons with total degree *k* < 200 are sparse. (***C***) The rich-club curves with different common neighbor coefficients *a*_Γ_. Inset: the maximum rich-club coefficient between k = 500 and k = 600 are positively correlated with the common neighbor coefficient. Both motif and rich-club results are calculated for a circular region in the network containing 2000 excitatory neurons to avoid artefacts from the periodic boundaries.

To demonstrate the presence of overrepresented pair and triad motifs in our model as found in the layer 5 of rat visual cortex [5], we first compare the number of bidirectional connections against Erdős-Rényi (E-R) random networks. We find significantly more bidirectional connections than expected in E-R networks (mean ± SEM: 1.94 ± 0.02, *p* < 0.001, one sample t-test), indicating that the bidirectional connections are overrepresented. A triad motif can be arranged in thirteen ways with all three neurons connected to each other (Fig 2*A*). As in [5], we compare the numbers of these motifs in our network against those in a null model, which is a random network with the unidirectional and bidirectional connection probabilities specified separately to match the original network. As shown in Fig 2*A*, the triad motifs in our model exhibit two salient features. First, for the most overrepresented motif, No.13 (the fully connected motif), its count relative to the null model (4.96 ± 1.86; *p* < 0.001, one sample t-test) is dominantly higher than the other motifs; this feature of the fully connected motif in our network model is consistent with measured data [5]. Second, the majority of the motifs that form a closed loop (ignoring direction) are overrepresented (except motif No.7 and No.11, Fig 2*A*); this is also consistent with experimental observations [5]. The existence of such closed loops in a triad motif can be characterized by its directed clustering coefficient (CC). The CC of a node (neuron) indicates the tendency of the node’s neighbors to cluster together [27], with its value ranging from 0 (none of the neighbors are connected to each other) to 1 (all neighbors are mutually reciprocally connected); the CC of a motif is obtained by averaging the CC of its three nodes. As shown in Fig 2*A*, the motifs with a closed loop have non-zero CCs and vice versa. As will be shown in the later sections, the CC is a structural property that has significant impact on neural dynamics.

In our model, the overrepresentation of the triad motifs with non-zero CC can be mainly explained by the distance-dependent connectivity, but the common neighbor property also makes a small yet significant contribution. To illustrate this, we first investigate how the change of the decay constant of the exponential connection probability function affects the motifs; we find that when it is decreased from 12 to 4, the number of motifs with non-zero CC relative to the null model increases (*r* = 0.82; *p* < 0.001). Aside from this spatial mechanism, we find that, a stronger common neighbor effect, as captured by a common neighbor coefficient *a*_Γ_ increased from 1 to 3, also increases the number of motifs with non-zero CC value (*r* = 0.20; *p* < 0.001). These results are consistent with the observation from a recent *in silico* study that the overrepresented triad motifs cannot be fully captured by the distance dependence and complementary mechanisms are needed [10]; our modeling study suggests that the common neighbor property can serve as such a mechanism.

#### Emergence of rich-club connectivity

We next show that our model gives rise to a rich-club connectivity phenomenon, and can explain the emergence of such a higher-order connectivity pattern. For this purpose, we calculate the normalized rich-club coefficient Φ(*k*), which is the ratio between the number of connections among neurons whose total degrees (i.e. the sum of in-degrees and out-degrees) are larger than *k* and that expected from random networks with the same in- and out-degrees for each neuron [30, 31]. In other words, this coefficient Φ(*k*) measures the extent to which the neurons with total degrees > *k* in a network are over-connected to each other. In our network, Φ(*k*) is consistently greater than 1.30 for neurons with *k* > 500 (Fig 2*B* blue). This result thus indicates that these neurons are over-connected to each other with at least over 30% more connections than expected from their in- and out-degrees, forming a densely connected rich-club. To further characterize these rich-club neurons, we calculate the dynamical importance for each neuron, defined as the fractional change in the largest eigenvalue of the adjacency matrix of the network upon removal of the neuron from it [32, 33], and find that rich-club neurons have higher dynamical importance (Fig 2*B* red). The heterogeneity of connection is also evidenced in the effectivity connectivity. To demonstrate this, we use the methods in [16, 34] to calculate the transfer entropy-based effective connectivity in our model, and find that the distribution of effective connection strengths is lognormal and there exists rich-club connectivity (see S1*A* and S1*B* Fig).

The emergence of such a rich-club connectivity structure in our model is dependent on the weakly correlated broad in- and out-degree distributions as well as the common neighbor property [3]. The former gives rise to a small group of well-connected “hub” neurons characterized by their large total degrees, and the latter makes those hub neurons more connected to each other than expected by chance. More specifically, we find that when the correlation *ρ*_*K*_ between in- and out-degrees or the hybrid parameter *q* (which controls the broadness of the in- and out-degree distributions; see Materials and methods) increases, the standard deviation of the total degree distribution also increases (*r* = 0.84 and *r* = 0.99, respectively; both *p* < 0.001). The common neighbor property increases the connection probability between a pair of hub neurons with large total degrees, because they are likely to share more common neighbors simply as they have more neighbors than those less well-connected neurons in the first place; indeed, we find that, as the common neighbor coefficient increases, the corresponding maximum normalized rich-club coefficient also increases (Fig 2*C*; *r* = 0.24, *p* < 0.001).

Rich-clubs have been found in macroscale human brain networks [31] and in the effective connectivity networks of mouse somatosensory cortex [16]. Very recently, at the synaptic level, rich-clubs have been found in a biologically constrained *in silico* model [10]. The rich-club behavior predicted by our circuit model is consistent with this line of research. Our model further suggests that the broad in- and out-degree distributions and the common neighbor factors are essential for the emergence of rich-clubs at the synaptic level, which could be tested in future experimental studies. Moreover, as illustrated in the following sections, the rich-club connectivity is related to heterogeneous neural dynamics as found in [12].

### Spatiotemporal neural dynamics

**Fig 3:**
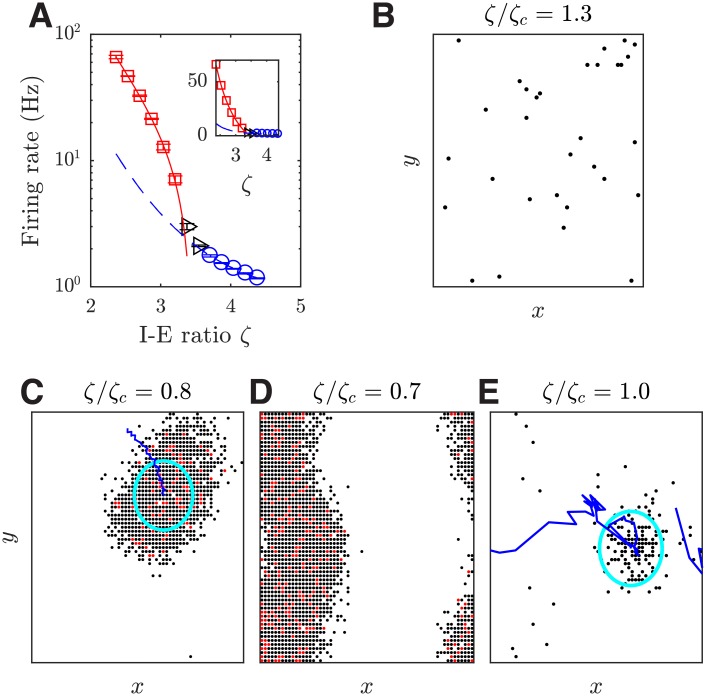
Transition of the circuit activity states induced by changing the I-E ratio *ζ*. (***A***) Mean firing rate of the excitatory population shows a phase transition around *ζ* = *ζ*_*c*_ = 3.375. The red solid line and blue dashed line are the two power functions fitted to the data points marked by red squares and blue circles, in the form of $a_{1}x^{b_{1}}+c_{1}$ and $a_{2}x^{b_{2}}$, respectively. The fitted coefficients are *a*_1_ = 25.47 ± 5.17 (0.95% confidence bounds), *b*_1_ = −3.535 ± 0.392, *c*_1_ = −23.72 ± 6.10, *a*_2_ = 2.199 ± 0.045, and *b*_2_ = −2.455 ± 0.116. The black triangles are the data points without fitting. The error bars show one SEM. Inset: the same plot but with the y-axis on a linear scale. (***B-E***) Snapshots of spiking patterns emerging from our local cortical circuit model with different I-E ratio *ζ* values. Black dots denote that one spike has been emitted from the excitatory neuron during a 5 ms period, and red dots denote two spikes. The cyan circles in (C) and (E) show one standard deviation of the fitted Gaussian firing rate profile and the blue curves show the trajectory of the centers of the pattern in the previous 40 ms. For the state shown in (E), the pattern appears intermittently and exhibits jumping behavior with a variable propagation trajectory.

Our spatially extended, heterogeneous circuit model exhibits a repertoire of states with rich spatiotemporal dynamics, beyond synchronous and asynchronous states that have been the main focus of previous studies [9, 19, 22]. To study the circuit dynamics, we systematically vary the I-E ratio *ζ*; neurophysiologically, this ratio could be controlled *in vivo* by non-competitive GABA receptor antagonists [35]. When *ζ* is gradually decreased, i.e. when excitation is much stronger relative to inhibition, the firing rate gradually increases. As shown in Fig 3*A*, the firing rate can be well fitted by two power functions, one for *ζ* < *ζ*_*c*_ (adjusted coefficient of determination *R*^2^ = 0.998), and another for *ζ* > *ζ*_*c*_ with a much larger exponent (Fig 3*A*; *R*^2^ = 0.988), where empirically *ζ*_*c*_ = 3.375. This change of firing rates indicates changes in the collective dynamic states of the circuit.

#### Transition from the asynchronous state to the propagating wave state

By studying the spatiotemporal activity patterns emerging from our local cortical circuit model, we next illustrate that the change identified in firing rates (Fig 3*A*) is related to a change from an asynchronous firing state to propagating wave states. We find that when the I-E ratio *ζ* is large (*ζ* > *ζ*_*c*_), the circuit does not show any structured patterns in spiking activity (Fig 3*B*); instead, it exhibits an asynchronous state. In this state, the globally averaged pairwise spike train correlation coefficient and the coefficient of variation of inter-spike intervals of the excitatory population are 0.002 ± 0.001 and 0.784 ± 0.002, respectively, which are similar to those found in the irregular and asynchronous firing state of randomly connected, balanced networks, e.g. [19, 36, 37].

On the other hand, when the I-E ratio *ζ* is small (*ζ* < *ζ*_*c*_), coherent patterns emerge from the circuit, in the form of a localized propagating wave (Fig 3*C* and S1 Video; see Materials and methods for pattern detection methods), or a global plane wave if *ζ* is further decreased (i.e. if the circuit is further disinhibited; Fig 3*D* and S2 Video); these patterns propagate across the neural circuit with relatively smooth and regular moving trajectories. Such global propagating waves with regular dynamics emerging from our model due to disinhibition are largely consistent with empirical studies reporting that when cortical circuits were disinhibited by using bicuculline to block GABA receptors, similar epileptiform global plane waves were observed [38]. Such spontaneous, regular waves arising due to disinhibition are thus fundamentally different from transient propagating waves as observed in the visual cortex of behaving monkeys [39] and in the MT area of anesthetized marmosets [40, 41].

When the circuit is around the transition point between the asynchronous state and the localized propagating wave state (*ζ* ≈ *ζ*_*c*_), spatially coherent patterns emerge intermittently from the asynchronous background activity (Fig 3*E*; Bayes factor > 10^2^, see Materials and methods). Such wave patterns have complex dynamics; they propagate coherently for a while (with a mean duration of 16.5 ± 1.3 and 10% duration values larger than 45.0 ms), and then jump to a different location (Fig 3*E*; see S3 Video). It is interesting to note that such propagating localized patterns have been found in cortex under normal excitability conditions, and their jumping behavior as observed in our model has been explicitly documented in [13]; similar intermittent wave patterns have also been observed in the cortex of awake rabbits [42]. Note that spikes in our model are sparse (typically under 6 Hz), consistent with the spontaneous firing rate of cortical circuits *in vivo* [43, 44]; this thus gives rise to the propagating pattern that is spatially sparse.

**Fig 4:**
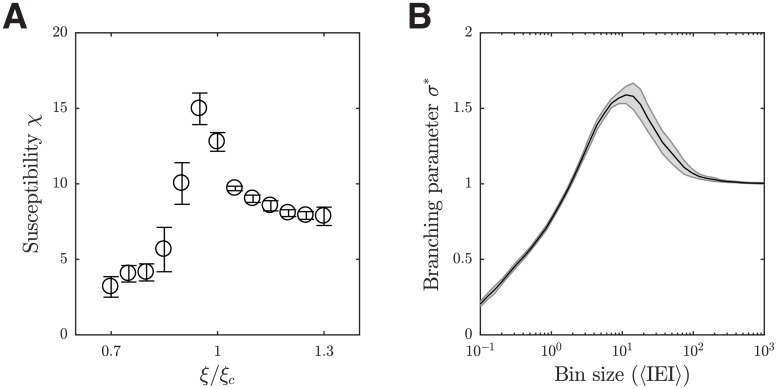
Near critical dynamics around the transition of the circuit activity states. (***A***) Susceptibility of the excitatory population at different I-E ratios *ζ*. 1 ms time bin is used for the calculation; the results are not sensitive to the choice of bin size. The error bars show one SEM of 10 trials. (***B***) Branching parameter σ* under subsampling as a function of the normalized bin size at *ξ*/*ξ*_*c*_ = 1. The shaded area shows one SEM of 10 trials.

We proceed to investigate the relationship between the transition state identified in our local circuit model and near-criticality dynamics. As in [45], we calculate the susceptibility *χ* = 〈*ρ*^2^〉 − 〈*ρ*〉^2^, where 〈⋅〉 denotes averaging over time, and we use the faction of active excitatory neurons in the model for the density of activation *ρ*. As shown in Fig 4*A*, the susceptibility is the largest in the transition region from the asynchronous to the localized propagating wave state of our model. We also calculate the branching parameter σ* of spikes of excitatory neurons, which is the average ratio between the number of spikes in one bin, divided by the number of spikes in the previous bin, with different bin sizes as in [46]. It has been shown that with the bin size normalized by the averaged inter-event interval 〈IEI〉 (the inverse of population firing rate), the curves of branching parameter σ* versus the normalized bin size are very similar across species and experiments (see Fig. 7*C* in [46]). At the transition point of our model (as shown in Fig 4*B*), the curve of branching parameter σ* (subsampling 100 neurons as in [46]) is quantitatively comparable to that reported in [46]. In particular, the maximum of branching parameter σ* under subsampling in our model is 1.61 ± 0.06, which is close to that of 1.4 in [46]. These results thus indicate that the transition state in our model is near criticality.

#### Theoretical analysis

**Fig 5:**
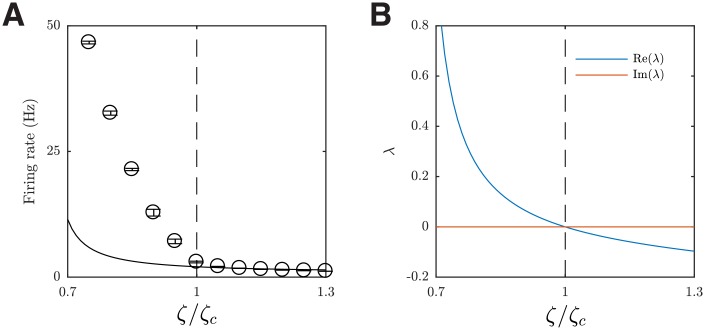
Stability analysis of the homogeneous, asynchronous state of the spatially-extended spiking circuit. (***A***) Mean firing rate of the excitatory population in the simulation of our local cortical circuits (dots) and the rate of spatially uniform activity from the analysis (solid line) with the optimal correction factor. The dashed line shows the critical point *ζ* = *ζ*_*c*_. (***B***) Eigenvalues with the largest real parts of the network’s dynamics in response to spatially periodic perturbations (spatial Fourier mode **k** = (1, 1)), with different I-E ratios *ζ* and the optimal correction factor. The eigenvalues in this parameter regime are real, with positive values emerging as the I-E ratio *ζ* decreases below the critical point *ζ*_*c*_ (dashed line), indicating a Turing bifurcation. The dominant spatial eigenmode has wave vector **k** = (1, 1), indicating that a single pattern is formed, consistent with that observed in the full spiking circuit.

To understand the mechanisms underlying this transition from the asynchronous state to the localized propagating wave state, we employ an analytical framework known as the diffusion approximation [47, 48], in particular, its recent extension for analyzing spatially extended, spiking neural circuits [49] (see S1 Appendix for details). Unlike more abstract models commonly used for studying emergent spatial patterns in neural media, this analytical approach preserves most of the biophysical details of the spiking neurons (Eq 4). We find that when the mean E-to-E synaptic strength used in the analysis is scaled by an empirical factor *h* = 0.4775, the critical I-E ratio *ζ*_*c*_ from the analysis and the simulation coincide; the role of *h* is to compensate for the simplifications imposed by the diffusion approximation (see S1 Appendix for details). As shown in Fig 5*A*, for *ζ* > *ζ*_*c*_, the mean firing rate of the spatially uniform activity predicted by the analysis, which corresponds to the asynchronous state, matches closely to our full spiking circuit model; however, for *ζ* < *ζ*_*c*_, the circuit’s mean firing rate becomes larger than that predicted by the analysis, as a sharp transition to spatially coherent pattern occurs in the full circuit model. We analyze the stability of the spatially uniform activity under spatially periodic perturbations, and find that it is stable for *ζ* > *ζ*_*c*_ but unstable for *ζ* < *ζ*_*c*_. In the latter case, we find that the formation of spatially coherent pattern is due to a Turing bifurcation (Fig 5*B*). Our analysis thus provides an explanation for the formation of the spatially coherent pattern in our cortical circuit model.

#### Common neighbor property of neural connectivity can induce the change of dynamic states

**Fig 6:**
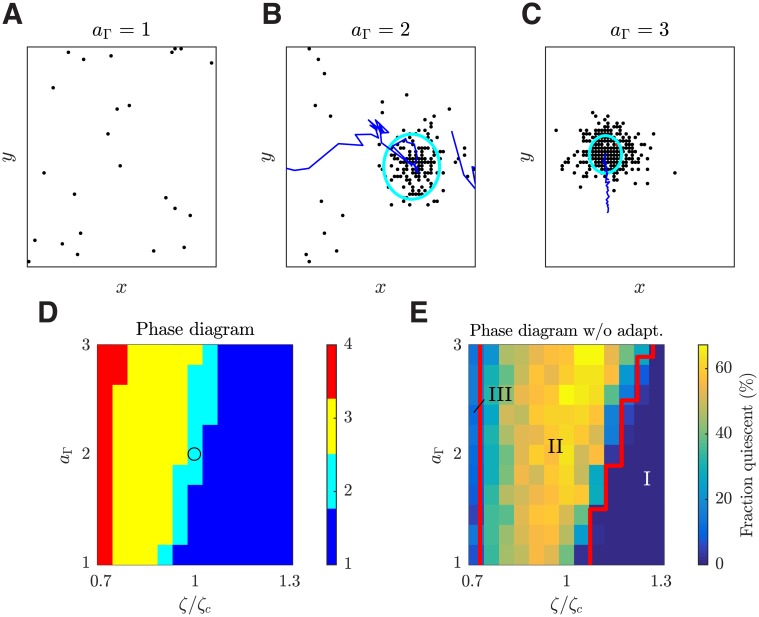
Common neighbor coefficient *a*_Γ_ can induce the transition from the asynchronous state to the localized propagating wave state. (***A-C***) Snapshots of spiking patterns in the local cortical circuit model with different common neighbor coefficient *a*_Γ_ values. These patterns are visualized in the same way as in Fig 3*B*–3*E*. (***D***) Phase diagram constructed based on the pattern detection rate (the percentage of time frames where a localized wave is detected) and the average firing rate of the excitatory population. The States 1, 2, 3 and 4 correspond to the asynchronous state, the transition state, the localized propagating wave state and the global plane wave state, respectively. The circle denotes the working point of our local cortical circuit model. (***E***) Phase diagram as in (*D*) but with the spike frequency adaptation removed, where the different states are shown by red boundary lines. The state I, II, and III correspond to the asynchronous state, the state with localized activity patterns that barely propagate (see S4 Video), and the global plane wave state, respectively. The excitatory neurons without firing a spike during the 10 second simulation time are detected as quiescent neurons.

We now demonstrate that the common neighbor factor provides a novel structure-based mechanism for shifting cortical activity states. We vary the common neighbor coefficient *a*_Γ_; a larger *a*_Γ_ means that connections will more likely be formed between neuron pairs sharing more common pre-synaptic neighbors (see Materials and methods). Note that the value of *a*_Γ_ does not affect the number of connections or the total incoming connection strengths of individual neurons. Instead, a larger *a*_Γ_ results in a more clustered network (i.e. with more triad motifs that have closed loops), as indicated by an increasing mean network CC (Fig 1*D*). We find that such a change in the connectivity induces a similar transition from the asynchronous state to the localized propagating wave state. Networks with smaller *a*_Γ_ and thus smaller CC values fire asynchronously and irregularly (Fig 6*A*); on the other hand, networks with larger *a*_Γ_ and CC values exhibit localized propagating patterns with regular moving trajectories (Fig 6*C*). Propagating patterns with complex dynamics, including variable propagation direction, speed, and jumping behavior, thus emerge from the network with intermediate CC values (Fig 6*B*); this scenario is largely consistent with the recent *in vitro* finding that moderately clustered networks appear optimal for initiation and propagation of diverse, complex propagating wave patterns [50].

These results reveal that both the neurophysiological mechanism (i.e. the I-E ratio *ζ*) and the structure-based mechanism (i.e. the common neighbor coefficient *a*_Γ_) can shift circuit activity state from the asynchronous to the propagating wave state. We thus use these two parameters (*ζ* and *a*_Γ_) to systematically explore the circuit dynamics based on the distinctive dynamic states we have found above. As shown in the phase diagram (Fig 6*D*), the transition from the asynchronous to localized propagating wave state can be induced across a range of the parameter values. We define a working point of the cortical circuit model at one such critical transition of the states with *ζ* = *ζ*_*c*_ and *a*_Γ_ = 2 (Fig 6*D*), where the average firing rate (3.0 ± 0.2) of the excitatory neurons are close to that found during spontaneous activity *in vivo* [43, 44].

#### Effect of spike-frequency adaptation

We now demonstrate that the spike-frequency adaptation (Eq 5) affects the spatiotemporal dynamics of our local cortical circuit model. For this purpose, we investigate neural circuit dynamics after removing the spike-frequency adaptation from our model (by setting Δ*g*_*k*_ = 0 in Eq 5). As shown in Fig 6*E*, spatiotemporal patterns can still be formed without adaptation, including localized activity patterns (State II) and global plane waves (State III). However, the localized activity patterns in State II have different dynamics, which are pinned to certain locations without clear long-range propagations as found in the original model with adaptation. This behavior of the localized patterns results in the scenario that within this state, only a small portion of excitatory neurons fire and the rest is quiescent. As in [9], we calculate the fraction of excitatory neurons quiescent in our spatially extended, heterogeneous network and find that this fraction is mostly greater than 50% for this state. In contrast, for the original network with the adaptation, the fraction of neurons quiescent is low (< 0.1%) for all the states. This result is consistent the observation that the inclusion of adaptation can reduce the fraction of neurons quiescent in randomly connected neural circuits with heterogeneous degree distributions [9]. In comparison, the removal of adaptation has less effect on the asynchronous state (State I in Fig 6*E*); the part of the asynchronous state in the original phase space with lower firing rates (Fig 6*D*; bottom right), after the removal of adaptation, remains as the asynchronous state with dynamics similar to those found in [37]. This is expected because in this region, the inter-spike intervals of individual neurons are rarely shorter than or comparable to the decay constant of potassium current (*τ*_*K*_ = 80 ms; see Eq 5), rendering the effect of spike-frequency adaptation negligible. Note that both an increase in the I-E ratio *ζ* in the circuit without spike-frequency adaptation and the introduction of adaptation can induce the transition from the state with localized activity patterns that barely propagate to the asynchronous state; this suggests that both factors tend to reduce excitation in the circuit. However, only the introduction of adaptation can induce the transitions from local to propagating patterns. This happens because the adaptation destabilizes the local pattern and fluctuations of the network would cause it to propagate away from its original location, forming a propagating wave.

### Dynamic working regime of spatially extended, heterogeneous cortical circuits

We next demonstrate that around the working point (i.e. the critical transition from the asynchronous to the localized propagating wave state), our model can quantitatively reproduce a great variety of key experimental findings regarding spatiotemporal dynamics of spontaneous neural activity. In addition, this critical regime further reveals how these dynamics are correlated to the connectivity structure.

#### Emergent, tightly balanced excitation and inhibition

**Fig 7:**
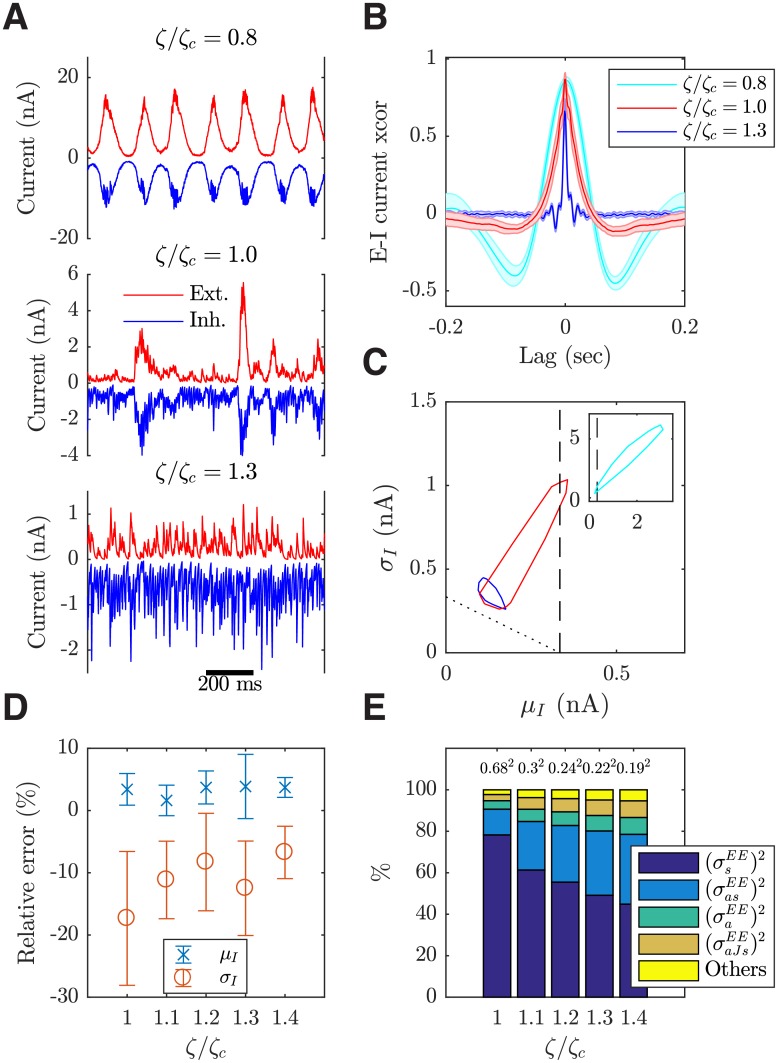
Properties of synaptic inputs to individual neurons in the local cortical circuit model. (***A***) Time series of total excitatory and inhibitory inputs received by individual excitatory neurons over 1 s in the different dynamic states (with *ζ*/*ζ*_*c*_ = 0.8, 1.0, 1.3 corresponding to the propagating wave state, the transition state and the asynchronous state, respectively; see Fig 3). (***B***) Average cross-correlation (xcorr) between the total E and I currents into excitatory neurons in the different dynamic states. The shaded area shows one SEM of 10 trials. (***C***) The convex hulls of the mean and standard deviation data points (pooled from 10 trials) of the total input current into individual neurons in the different dynamic states, denoted by the same color-coding as in (B). The region to the left of vertical dashed line is where the mean input current is less than the threshold current. The region above the inclined dotted line is where the mean plus one standard deviation of the input current is above the threshold current. (***D***) The relative errors of estimating the total standard deviation *σ*_*I*_ (and mean *μ*_*I*_) of the recurrent currents with the sum of individual components as given by the variance decomposition (Variance decomposition analysis), averaged over the excitatory neurons and over a period of 50 ms within each trial. (***E***) The contributions to the variance in recurrent currents from various sources are shown across circuits with different I-E ratio *ζ*. The numbers above the bars are the corresponding total variances averaged over excitatory neurons (nA^2^).

It has been found *in vivo* that cortical neurons receive strongly correlated excitatory and inhibitory inputs, with the former closely tracked by the latter as characterized by a strong cross-correlation around zero time lag [23, 51]; such tight proportionality between E and I inputs is regarded as a tight balance [52]. We now show that tightly balanced synaptic inputs with the dynamical properties as found in [23] emerge from the working point of our model as defined above; the balanced condition in our current model is thus referred to as such a tightly balanced one. Fig 7*A* (middle panel) shows that the magnitudes of E and I currents into a neuron closely track each other in time, with strong cross-correlations around zero time lag (Fig 7*B* red), which decay to zero within around 50 ms; note that this decay time is largely consistent with that measured in *in vivo* recordings (see Fig. 1 of [23]). It is also interesting to note that only near the critical transition state, as shown in Fig 7*A* (middle panel), synaptic inputs appear to exhibit large, occasional excursions in amplitudes; such bumpy properties of synaptic inputs have been explicitly documented in [23]. When the system is in the asynchronous state, the inputs do not have such bumpy properties (Fig 7*A* bottom panel) but the E and I currents are still highly correlated at zero time lag. However, the cross-correlation decays to zero much faster (within around 15 ms; Fig 7*B* blue) than that measured in [23]. In the propagating wave state, the strong cross-correlation is also present (Fig 7*B* cyan) but the synaptic inputs become semi-periodic and regular (Fig 7*A* top panel), apparently inconsistent with the large fluctuations observed in synaptic inputs *in vivo*.

Another key property of the synaptic inputs to cortical neurons is that they keep neurons in a high-conductance state [24, 53]. We now demonstrate that in our conductance-based model, the statistical properties of such a high-conductance state can be captured. At the working point, the mean excitatory synaptic conductance into individual excitatory neurons is of the order of the leaky conductance (with the ratio between them being 1.27 ± 0.15; 400 sample neurons pooled from 10 trials), as found in pyramidal neurons in areas 5, 7, and 21 of the parietal cortex of awake cats [24]. Furthermore, the mean and fluctuation (i.e., SD) ratios between inhibitory and excitatory conductances (2.81 ± 0.21 and 2.95 ± 0.59, respectively) are also consistent with those measured in [24]. As a result of such synaptic inputs, the membrane potential is depolarized (with a mean of −61.7 ± 0.6 mV) and exhibits large fluctuations (with a SD of 4.1 ± 0.3 mV). However, our model does not capture the observed decrease in inhibitory conductance within 20 ms before a spike [24]. As the I-E ratio becomes larger (*ζ* > *ζ*_*c*_), and mean excitatory conductance is still comparable to the leaky conductance (with a ratio of 0.79 ± 0.03 at *ζ*/*ζ*_*c*_ = 1.3, in the asynchronous state); however, when the circuit becomes disinhibited with a smaller I-E ratio (*ζ* < *ζ*_*c*_), the mean excitatory conductance becomes pathologically high (6.67 ± 0.68 times the leaky conductance at *ζ*/*ζ*_*c*_ = 0.8, in the propagating wave state).

#### Structural heterogeneities contribute to neural fluctuations

Similar to the conductances, the excitatory and inhibitory input currents into individual neurons also show large fluctuations (Fig 7*A*). This dynamic regime near the working point of our model is fluctuation-driven [54]; that is, the mean input currents into individual excitatory neurons are subthreshold as a result of the tight balance described above and, without the time varying fluctuations around their mean input, the neurons would not fire (Fig 7*C* red). In this regime, the neural dynamics are largely determined by the variances in the recurrent currents and therefore highly variable, as characterized by a mean coefficient of variation of inter-spike intervals of the excitatory neurons at 0.93 ± 0.03, consistent with that measured from the spontaneous activity *in vivo*. In the asynchronous state (*ζ* > *ζ*_*c*_), the mean input currents remain subthreshold (Fig 7*C* blue); on the other hand, in the disinhibited propagating wave state (*ζ* < *ζ*_*c*_), the mean input currents become superthreshold (Fig 7*C* cyan).

By incorporating a range of realistic features of cortical connectivity into the spiking circuit model, such as the heterogeneities in both connection topology and strength, our model allows us to explore their effects on the variable neural dynamics. In particular, we investigate how the structural heterogeneities contribute to the variances in the recurrent excitatory currents in the fluctuation-driven circuits with *ζ* ≥ *ζ*_*c*_. For this purpose, we develop an analysis method called variance decomposition analysis (see Materials and methods). As described in Eq 9 syn, there are four components determining the recurrent currents, namely, the connection topology, the connection strength, the pre-synaptic dynamics and the postsynaptic dynamics, denoted by the shorthand letters *a*, *J*, *s* and *V*, respectively. Our analysis shows that there are 15 different sources of variance of the recurrent currents (Variance decomposition analysis), arising from each of the four components (*a*, *J*, *s* and *V*) contributing via either its own (co-)variance or mean value (see Materials and methods). For example, one such source arises from the coupling among the variances of *s* & *a* and the mean values of *V* & *J*; we denote this source by ${\left({\hat{\sigma }}_{as}^{EE}\right)}^{2}$, only including in the subscript the letters representing those components contributing via their (co-)variances. In our variance decomposition analysis we assume that the four components are mutually independent and ignore the moments beyond second-order. To validate this analysis, we study how well the sum of individual variance sources (RHS of Variance decomposition analysis) matches the total variance (LHS of Variance decomposition analysis) using numerical data with different I-E ratio *ζ*. As shown in Fig 7*D*, the two match with reasonable accuracy.

The contributions of these 15 sources across fluctuation-driven systems with different I-E ratios are shown in Fig 7*E*; we find that, to capture most of the variance in recurrent currents across different I-E ratios, at least four sources need to be considered, including ${\left({\hat{\sigma }}_{s}^{EE}\right)}^{2}$, ${\left({\hat{\sigma }}_{as}^{EE}\right)}^{2}$, ${\left({\hat{\sigma }}_{a}^{EE}\right)}^{2}$ and ${\left({\hat{\sigma }}_{aJs}^{EE}\right)}^{2}$. Note that the variances in connection topology (*a*) and strength (*J*) contribute to 3 out of 4 of the major sources; as shown in Fig 7*E*, without these two structural factors, at *ζ* = *ζ*_*c*_, only 78.3 ± 9.9% of the total variance can be captured by ${\left({\hat{\sigma }}_{s}^{EE}\right)}^{2}$ alone. Therefore, the variances of connection topology and strength make a significant contribution to the variable recurrent currents at the working point of our model. When the I-E ratio increases to *ζ*/*ζ*_*c*_ = 1.4, the amount of variance captured by ${\left({\hat{\sigma }}_{s}^{EE}\right)}^{2}$ alone further decreases down to around 50%. This result thus reveals the importance of the structural heterogeneities in generating the variable, fluctuation-driven neural dynamics, going beyond previous studies about variable neural dynamics, in which such variances in connection topology and strength were not captured, e.g. [19, 36, 47].

#### Emergent heterogeneous population coupling

Recently, it has been found that in the deep layers of sensory cortex of awake mammals, individual neurons correlate (couple) to the population activity differently, ranging from strongly correlated “choristers” to weakly correlated “soloists” [12]; this experimental study has revealed a fundamental relationship between individual neurons and a larger population. However, the dynamic nature of this diverse coupling behavior and how it is related to structural connectivity remains unclear. We now demonstrate that such diverse coupling can emerge in our model near the critical transition from the asynchronous to the localized propagating wave state. In addition, we illustrate that these heterogeneous neural dynamics are related to the rich-club connectivity predicted by our model.

**Fig 8:**
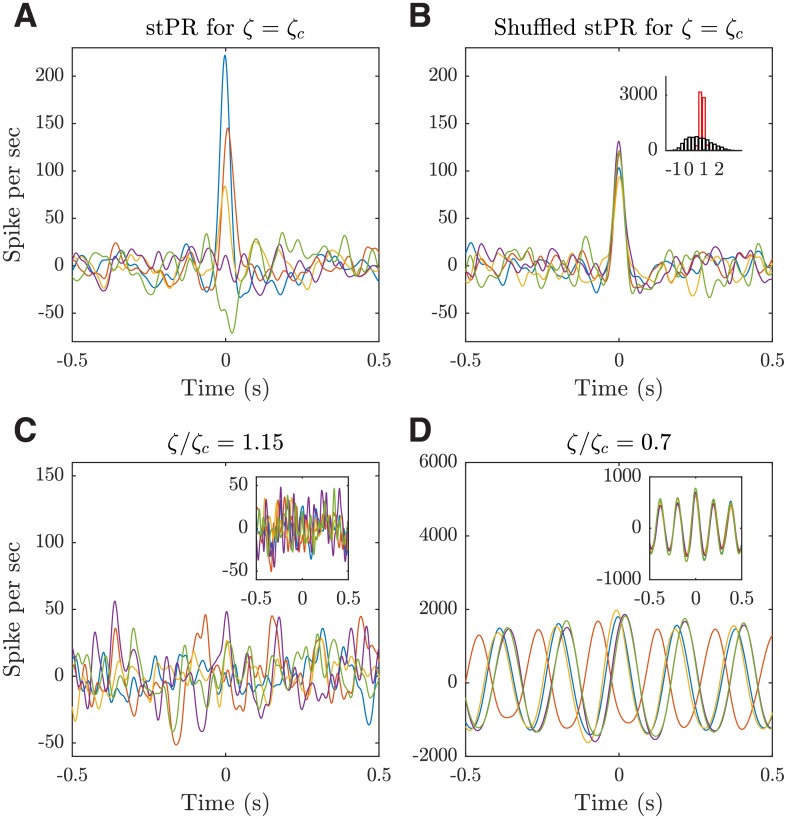
Emergent, heterogeneous population coupling in the dynamical working regime of the local cortical circuit model. (***A***) Spike-triggered population rate (stPR) for five representative neurons in a single trial. (***B***) stPR for the same five neurons but with spikes shuffled. Inset: distributions of stPR coefficients pooled from 10 trials before (black) and after (red) spike shuffling. The coefficients are defined as the stPR values at zero time lag normalized by the median value of the shuffled data. For each trial, 10 samples of 66 excitatory neurons are randomly chosen to match the sample size used in experimental studies [12]. Neurons are sampled from a circular area with a radius of 25 grid units (about 125 ¼m) for approximating the spike detection range of a single shank of silicon electrode array used in [12]. The results are not sensitive to the size of the sampling area or the number of sampled neurons. (***C-D***) stPRs for *ζ* = 1.15 and *ζ* = 0.7, respectively. Insets: shuffled stPRs. For *ζ* = 1.15, the model is shifted away from the working regime into the regular propagating wave state; while for *ζ* = 0.7, it is shifted into the irregular, asynchronous state.

To quantify the population coupling in our model, we calculate the spike-trigged population rate (stPR); as in [12], this is done by first summing the spike train of mutiple neurons to get the population rate, and then correlating the spike train of each neuron with this population rate. As shown in Fig 8*A*, the neurons in our model exhibit diverse population coupling behavior; that is, there are neurons with strongly positive, weakly positive, neutral or even negative correlations with the population activity. To test the significance of this diverse coupling, we use the method in [12] to shuffle the spike data in a manner that preserves both the population rate and the average firing rate of each neuron but randomizes the timing of individual spikes. We find that, after the shuffling, the diverse population coupling behavior almost completely disappears (Fig 8*B*). For a quantitative comparison, we calculate the stPR coefficients as in [12]. In our model, the stPR coefficients before shuffling ranges from −0.96 ± 0.26 to 2.85 ± 0.23, with a mean of 0.81 ± 0.09 and a standard deviation of 0.76 ± 0.06; these results are comparable to those measured in the deep layers of S1 of awake mice, i.e. with a range from -0.5 to 2.5 and a mean around 0.5 [12]. After shuffling, however, the standard deviation of stPR coefficients becomes much smaller (0.14 ± 0.03; Fig 8*B* inset; *p* < 0.001, two-sample F-test), confirming that the diverse population coupling behavior emerging from our model is an inherent dynamical property of our local cortical circuit model.

We find that our model does not show such diverse population coupling when it is shifted away from the working point (i.e., the critical transition point between the asynchronous and the propagating wave states), although the structural topology remains the same. As shown in Fig 8*C*, in the asynchronous state, the stPRs are noisy and show little difference from the shuffled results. As the network becomes more disinhibited with a smaller *ζ* value, the stPRs become periodic due to the regular movement of patterns (Fig 8*D*), not consistent with those reported in [12].

**Fig 9:**
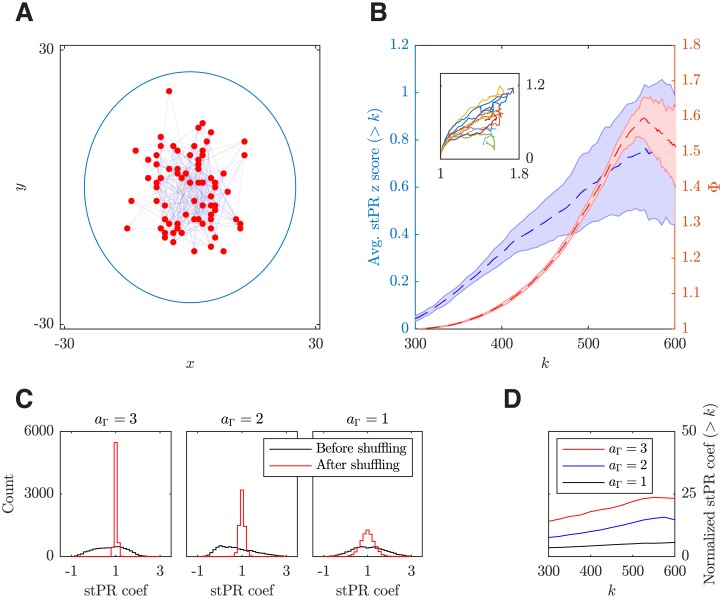
Rich-club connectivity is related to the heterogeneous population coupling. (***A***) An example of the rich-club connectivity among high total degree (*k* > 600) excitatory neurons (red dots). Only a 10% random subset of the synaptic connections are plotted (lines) for visualization purpose, with a probability of 1/Φ(*k* = 600) colored in blue and otherwise in yellow to illustrate the proportion between the number of connections expected from their total degrees alone (blue) and the number of extra connections due to the rich-club connectivity (green). The circle shows the range within which both the rich-club and stPR coefficients are calculated as used in Figs 2*B* and 8. (***B***) The rich-club neurons have high stPR coefficients. Left y-axis (blue): the blue dashed line shows the stPR coefficient z scores for the neurons with a total degree > *k* averaged over 10 trials and the shaded area shows one SEM. Within each trial, 10 random 66-neuron samples (as in Fig 8) are collected to calculate the *k*-dependent stPR coefficient z scores. Right y-axis (red): the red dashed line shows the normalized rich-club coefficient curve from Fig 2*B* and the shaded area shows one SEM. Inset: normalized rich-club coefficient Φ(*k*) (y-axis) versus *k*-dependent stPR coefficient z score (x-axis) from 10 realizations of the network (shown with 10 different colors). (***C***) Distributions of stPR coefficients pooled from 10 trials before (black) and after (red) spike shuffling from the local cortical circuits with different common neighbour factors *a*_Γ_. For comparison, the total count (area under curve) and bin size are the same for each distribution. (***D***) stPR coefficients before shuffling, normalized by the SD of stPR coefficients after shuffling (i.e., coefficients of control case) and plotted in the same *k*-dependent manner as in (*B*). Each curve shows the average of 10 trials.

To unravel whether and how the heterogeneous connectivity is related to this diverse coupling, we compare the rich-club connectivity of the neurons with their population coupling behavior. As mentioned above, the normalized rich-club coefficient Φ(*k*) characterizes the degree of “over-connection” among the neurons with a total degree higher than *k*; Fig 9*A* shows the connections among such a group of high degree neurons. In order to compare Φ(*k*) against the population coupling behavior, we need to quantify the corresponding degree of “over-coupling” to the population rate of the corresponding neurons (with a total degree higher than *k*). This can be achieved by calculating the average z-score of stPR coefficients for those neurons. As shown in Fig 9*B*, this *k*-dependent stPR coefficient z-score shows an overall increasing trend when the total degree *k* increases; this trend is closely followed by the normalized rich-club coefficient Φ(*k*). For a more direct comparison, we plot the coefficient z-scores against the corresponding normalized rich-club coefficients with the same *k* (Fig 9*B* inset), and confirm that the z-scores can be largely explained by Φ(*k*) (*r* = 0.84; *p* < 0.001).

To further illustrate the relationship between the rich-club connectivity and the diverse population coupling, we vary the common neighbour factor *a*_Γ_ that can modulate the rich-club coefficients (Fig 2*C*) while preserving the in- and out-degrees of individual neurons (see Materials and methods). We find that when the common neighbour factor *a*_Γ_ decreases from 3 to 1, the difference between the original distribution of stPR coefficients and the distribution of the control case (i.e., the case with random shuffling of spikes) gradually diminishes (Fig 9*C*), indicating that the diverse population coupling effect becomes less significant. This trend can be quantified by normalizing the original stPR coefficients with the standard deviation (SD) of those coefficients of the control case. These normalized coefficients are then compared across circuits with different values of *a*_Γ_; as shown in Fig 9*D*, the trial-average of such coefficients decreases as the common neighbour factor *a*_Γ_ decreases. As decreasing *a*_Γ_ results in a decrease in the peak value of normalized rich-club coefficient Φ(*k*) (see Fig 2*C*), these results indicate that the rich-club connectivity in our circuit model is closely related to the diverse coupling behavior of neural dynamics.

#### Dynamic propagating patterns and precise spiking structures

We next demonstrate that in our local cortical circuit model, propagating wave patterns emerging near the transition point (Fig 6) can quantitatively account for the dynamic properties of propagating spiking waves of UP states found in spontaneous activities of cortical neurons in layer 5 of awake rats [14]. In addition, our model can capture precise spiking structures, which have been found to coexist with propagating waves [14]. We further show that the spatial extension property of our model is essential for the emergence of these patterns.

**Fig 10:**
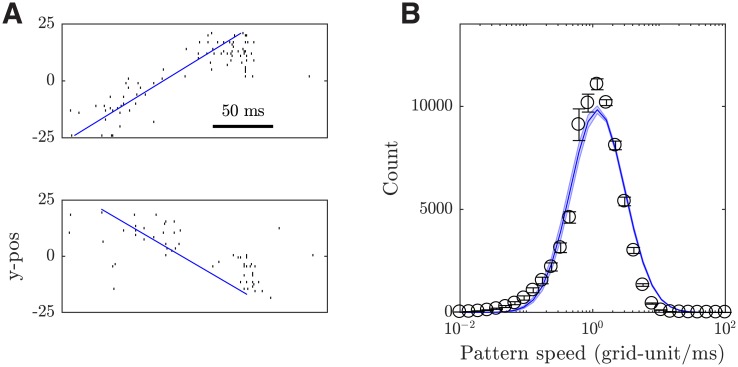
Dynamic properties of propagating wave patterns emerging in the dynamical working regime of the local cortical circuit model. (***A***) Raster plots of 50 neurons randomly sampled from a 10-by-50 grid-unit region, sorted according to their y-coordinates, showing two travelling patterns moving in opposite directions at different time points. The solid blue line is the linear fit to the first spikes at each y-position (*y* = 0 denoting the y-center of the sampling region). (***B***) The distribution of the pattern propagation speed (dots) with the log-normal fit (blue solid line). The error bars and shaded area show one SEM.

Around the working point (i.e. the transition point from the asynchronous to the localized propagating wave state), our model exhibits a propagating wave pattern with irregular trajectory and variable speed as illustrated above (Fig 3*E*). To compare these wave patterns with those found in [14], we visualize the firing activity using raster plots where neurons are sorted based on one spatial coordinate; Fig 10*A* shows a wave propagating with a component of its direction along this spatial coordinate. To characterize the pattern propagation in our model, we calculate the distance traveled every millisecond to obtain the propagation speed distribution. We find the propagation speed is highly variable, as evidenced by its log-normal distribution (Fig 10*B*). This propagation speed distribution has a mean of 1.72 ± 0.06 grid-units ms^−1^ (12.7 μm ms^−1^; see Materials and methods for the conversion) and 10% of values larger than 3.55 ± 0.11 grid-units ms^−1^ (26.3 μm ms^−1^), which matches the highly variable propagation speed measured in the cortex of awake rats with values spanning from 8 to 40 μm ms^−1^ [14]. Such variability in speed has also been found in cortical slices, where the propagation of localized excitation patterns often abruptly change speed; under normal excitability conditions, the typical speed values are between 6-10 μm ms^−1^ [13]. In our model, the variability of propagating wave patterns is lost when the system is disinhibited (with small I-E ratio *ζ*), as patterns propagate in a more regular way.

**Fig 11:**
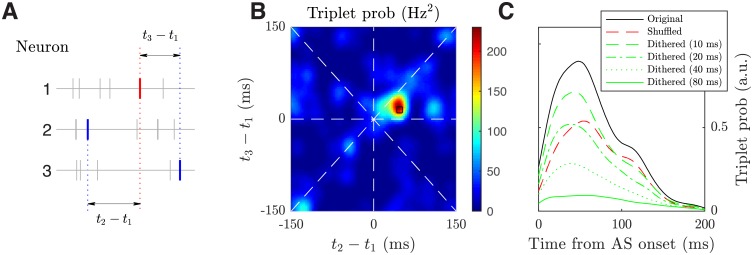
Emergent precise spiking structures. (***A***) Schematic of a spike triplet that is described by two inter-spike intervals. (***B***) The probability distribution of two inter-spike intervals from a representative neuron trio. The black square denotes the precisely repeating triplets occurring around ± 10 ms of the mode. (***C***) Occurrence of precisely repeating triplets peaks shortly after the start of activated states. The time from activated state (AS) onset is calculated based on the first spike of the triplet. The black, red, and green lines are the trial-averaged normalized probability density functions (PDFs) for the original data, shuffled data [14], and data dithered with different window sizes (method L in [56]), respectively. For each trial, 50 samples of 50 excitatory neurons are randomly chosen from a 10-by-50 grid-unit region (about 50-by-250 μm) to approximate the spike detection region of two neighboring shanks of silicon electrode array used in the experimental study by [14]. The results are not sensitive to the width of the rectangular sampling area. Within each sample, 50 neuron trios are randomly chosen to get the original and shuffled/dithered spike triplet counts.

Around the working point, our model can also capture the precise spiking structures during spontaneous activity in layer 5 of rodent somatosensory cortex [14]. Precisely repeating patterns embeded in variable neural activity have also been found in spontaneous activity with critical dynamics [55]. In our model, precise spiking structures can be detected by considering spike triplets. As illustrated in Fig 11*A*, a spike triplet is composed of three spikes, each from a different neuron, and can be characterized by the two inter-spike intervals. The joint distribution of the two inter-spike intervals should have a flat structure without any preferred mode if the three neurons fire randomly as independent stationary Poisson processes. Therefore, a statistically unlikely large peak in the joint distribution, as found in our model (Fig 11*B*), means a temporally precise, sequential firing pattern appears repeatedly in this neuron trio. Following the method in [14], the triplets with inter-spike intervals 10 ms around the mode are detected as precisely repeating triplets.

To further test the statistical significance of the detected precisely repeating triplets in our model, we first shuffle the individual spike timing using the method in [14] and compare the number of detected triplets before and after the shuffling, with respect to the time from activated state onset, which serves as an objective temporal reference for comparison. As shown in Fig 11*C*, the number of the detected triplets (or equivalently the triplet probability) is consistently lower after shuffling within 100 ms after the activated state onset, confirming that a significant portion of detected triplets in the original data are intrinsically structured. In particular, the triplet probability before shuffling peaks shortly after the activated state onset (around 48 ms; peak value 0.78 ± 0.50 times higher than that of the shuffled data; *p* < 0.001 for two sample t-test), which is consistent with the *in vivo* findings [14]. Additionally, we use the dithering method (method L in [56]) to test the statistical significance of this result. As shown in Fig 11*C*, the dithering method gives a characteristic decay of the triplet probability when the dithering window size increases from 10 ms to 80 ms (compared with the triplet detection window size 20 ms), and the peak triplet probability before dithering is significantly higher than that of the dithered ones (*p* < 0.006 for two sample t-test), confirming the statistical significance of the detected triplets [57].

The detected activated states largely correspond to a spiking wave pattern passing through the sampling region, as the chance of finding the center of a wave pattern within the sampling region during activated states is nearly three times (2.84 ± 0.62) as high as the average. Therefore, the results in Fig 11*C* indicate that the precisely repeating triplets are initiated by the propagating waves patterns, as found in [14]. In our model, when the I-E ratio *ζ* is shifted further into either the asynchronous state or the regular propagating wave state, no such statistically significant precise spiking structure can be detected. In [58], however, spike sequences can reliably arise in an irregular, asynchronous state of randomly connected neural networks with lognormal synaptic weight distributions, and these sequences are organized along the unidirectional synaptic pathways embedded by rare strong synapses.

In our local cortical circuit model, due to its spatial extension property formed by the distance-dependent connectivity, spikes of one group of neurons would result in those neurons that are physically proximal to them to fire, resulting in propagating patterns. To further dissect this role of spatial extension in our model, we set all of the decay constants of the exponential connection probability functions in our model $\tau_{D}^{\alpha \beta }\to \infty$, so that the connectivity no longer depends on the spatial locations of the neurons. This effectively removes the spatial extension property, while keeping all the other connectivity properties of our model fixed. We find that propagating patterns cannot be formed and precisely repeating triplets cannot be detected. These results thus demonstrate the importance of such a spatial extension arising from the distance-dependent connectivity in shaping complex cortical dynamics, indicating that structured spatiotemporal patterns can emerge from the highly variable firing activity of individual neurons in such spatially extended networks.

## Discussion

In this study, we have shown that by uniquely integrating the essential anatomical and physiological properties of local cortical circuits, our novel model exhibits a rich repertoire of activity states ranging from the asynchronous and the localized propagating wave states to the global propagating wave state, beyond the synchronous and asynchronous states that have been the main focus of previous studies [9, 19, 22]. As we have demonstrated, in the dynamical regime of the transition from the asynchronous to the localized propagating wave state, our model can reconcile an otherwise disparate set of key experimental findings on neural dynamics [11, 12, 14, 23, 24], and can relate these dynamics to the underlying connectivity structure. In addition, our results make novel predictions of cortical circuits. Our work thus provides a unified framework for understanding the organizational properties of anatomic connectivity, neural dynamics and their relations in cortical circuits.

### Integrated connectivity features and emergent, higher-order connectivity structures

We have developed a generative model to incorporate key connectivity features at the synaptic level into our local cortical circuit model; these features include distance-dependence [3, 4], the common-neighbor property [3], and heterogeneities in both numbers and strengths of neural connections [5, 6, 9, 10]. As we have demonstrated, the fundamental advantage of our generative model is that it not only recapitulates these connectivity features, but also accounts for the formation of higher-order connectivity structures such as overrepresented triad motifs. Overrepresented triad motifs as predicted in our model have been found in layer 5 of rodent visual cortex [5]; we have illustrated that both the distance-dependent property and the common neighbor factor contribute to the emergence of these motifs. This is consistent with a recent study of *in silico* local cortical circuits, in which it has been shown that overrepresented motifs cannot be fully captured by the distance dependent connectivity property and some additional mechanisms are needed [10]; our result suggests that the common neighbor factor can serve as such a mechanism.

In addition, our model predicts and explains the existence of rich-club connectivity at the synaptic level. As we have illustrated, the combination of two elementary connectivity properties, i.e. the heterogeneous in- and out-connectivity degrees and the common-neighbor property, is essential for the emergence of this connectivity behavior; the former gives rise to some well-connected hub neurons, and the latter makes them more connected among themselves than chance, as quantified by the correlation between the common neighbor and the rich-club coefficients. At the macroscopic level of connections between different brain regions, rich-club structures have been found [31, 59]. At the microscopic level, recent work has reported the existence of rich-club structures in the effective connectivity quantified by information transfer between neurons in mouse somatosensory cortex [16]; very recently, rich-club connectivity has been found in biologically constrained *in silico* reconstructed microcircuits [10]. Our findings are consistent with these previous reports, but go beyond them by identifying the elementary factors that contribute to the emergence of rich-club connectivity at the synaptic level and by relating such connectivity to a key dynamical property of cortical microcircuits, i.e. the diverse coupling behavior of individual neurons to the population as found in [12].

### Dynamical working regime of local cortical circuits

Unravelling the dynamical and functional implications of the connectivity structure of local cortical circuits is currently the focus of substantial scientific interest. For instance, clustered connectivity with stronger than average synapses has been studied to understand how it is related to the variability of neural spikes during spontaneous and evoked activity [20] and bistable dynamics [60]; in [61], [21] and [22], models with distance-dependent connectivity have been developed to study how this connection property affects cortical dynamics; the dynamical effect of heterogeneity in number of connections on cortical dynamics has been studied in [9] and [62]; the effect of heterogeneous connection strengths on stabilizing sparse neuronal activity and spike-based communications has been studied in [63] and [58], respectively. Rather than considering these connectivity properties in isolation as in these previous studies, for the first time our model integrates them into the same modeling framework to elucidate how a rich repertoire of cortical states can emerge; these include the asynchronous state, localized propagating wave and global propagating wave states, beyond the asynchronous state that has been the main focus of previous studies. To further explore these rich spatiotemporal dynamics and relate them to connectivity of cortical circuits at different scales, it would be interesting to extend our local cortical circuit model to incorporate cortical laminar structures [64] and to further incorporate realistic inter-areal connectivity features [65, 66].

Our work identifies a dynamical working regime of local cortical circuits, within which localized propagating wave patterns emerge intermittently from the asynchronous state and give rise to near critical network dynamics. Our work thus contributes to the growing line of research on critical network dynamics [67–69] from a novel perspective particularly by demonstrating that in this working regime with dynamical wave patterns, a great variety of key neurophysiological findings can be reconciled and can be related to the connectivity structure. As we have demonstrated, in this working regime, bumpy excitatory inputs tightly track the inhibitory ones with their cross correlation decaying to zero within 50 ms, as found in the barrel cortex of rats [23], and the conductance properties are consistent with those measured in [24]. As demonstrated in our study, the realistic variability of cortical connectivity including the broad in- and out-degree distributions, spatial dependence and common neighbor properties contributes significantly to the variability of neural spiking activity, thus relating the variability of neural dynamics to that of connectivity. In addition, we have identified a new anatomic mechanism (i.e. the common neighbor property) for altering the boundary of the circuit activity states. This anatomic mechanism predicts that an increase in the clustering of neural connectivity would gradually shift the circuit state from the irregular, asynchronous state to the state with regular propagating waves; this prediction could be tested by modulating protein kinase C to modify the degree of clustering in neural circuits as in [50].

Furthermore, we have shown that in this dynamical working regime of our model, individual neurons are correlated to the firing rates of the population in a diverse way, with some neurons (choristers) highly correlated to the population activity, while others (soloists) showing correlations that are smaller than expected by chances or even anti-correlations; this diverse coupling behavior has been found in deep layers of visual cortex of awake mice and monkeys [12], but cannot be reproduced in either the asynchronous or the regular propagating wave regime. By showing that the stPR coefficients are proportional to the rich-club coefficient and that reducing the degree of rich-club connectivity while preserving the in- and out-degrees of neurons would result in the decrease of the statistical significance of diverse population coupling, our results further establish a relationship between diverse coupling as found in [12] and the rich-club connectivity. This result provides another prediction regarding the organizational properties of cortical circuits.

In the dynamical working regime, our model exhibits localized propagating wave patterns with complex dynamics; such dynamical patterns appear intermittently and propagate across the circuit for a while and then jump to another seemingly random location, and their propagation speeds are very variable. Notably, such localized propagating patterns with jumping behaviour have been found in spontaneous activities of the slices of rat somatosensory cortex bathed in low Mg solution or under normal excitability conditions once a shock was applied to stimulate neurons [13]. The variable propagation speeds as found in our model are also consistent with those measured from layer 5 of somatosensory cortex of awake rats [14]. Propagating wave patterns with random initiation sites and propagation directions have also been observed during spontaneous activity in both the sensorimotor cortex of behaving mice [70] and the visual cortex of behaving monkeys by using voltage sensitive imaging recordings [39]. In our model, such dynamical wave patterns cannot be captured in the asynchronous regime, within which neurons asynchronously emit spikes without any structured patterns. If the circuit is shifted into the propagating wave states by decreasing the I-E ratio, the circuit exhibits more regular, global plane waves without variability in propagation speed; similar regular waves have been found in pharmacologically manipulated, disinhibited neural circuits [38]. The emergent dynamical wave patterns in the working regime of our model could communicate information within cortical circuits due to their propagation property. The rich dynamics of these patterns may enable such wave-based communication to be implemented in a fundamentally distributed way across space and time.

The identification of the working regime with dynamical wave patterns, which can uniquely reconcile a wide range of anatomical and physiological observations, represents a key advance toward understanding the working mechanism of cortical circuits. To experimentally test this dynamical regime, it would be ideal to combine imaging studies and massive-unit recordings to visualize and record neural activity at different levels, and to analyze recorded neural signals in conjunction with analysis of neural anatomy by using the same methods as in our modeling study.

## Materials and methods

### Generative connectivity model of local cortical circuits

We first describe the generative connectivity model used to construct the topological structure of the local cortical circuits. The algorithm of the generative model consists of two stochastic procedures. The first stochastic procedure synthesizes the three essential connectivity properties, i.e. the heterogeneous in- and out-degree distributions, the distance dependent connectivity, and the common neighbor property. The second stochastic procedure controls the relationship between connection topology and strength [26]. The details of the first procedure are as follows:

1. Individual neurons are embedded on a 2-dimensional plane with periodic boundary conditions. *N*^*E*^ excitatory neurons are located at integer coordinates and *N*^*I*^ inhibitory neurons are distributed uniformly randomly in the plane. The Euclidean distance $D_{ij}^{\alpha \beta }$ is calculated between each pair of neurons *i* from population *α* and *j* from population *β*, where *α* and *β* are either excitatory (E) or inhibitory (I). The connection probability between neurons is proportional to the distance-dependent factor $\Omega_{ij}^{\alpha \beta }$. Consistent with the findings in [3] and [4], this factor is modelled as an exponential function of $D_{ij}^{\alpha \beta }$; that is, $\Omega_{ij}^{\alpha \beta }=e^{-D_{ij}^{\alpha \beta }/\tau_{D}^{\alpha \beta }}$. For simplicity, we only incorporate heterogeneous features into the E-to-E subnetwork as in other modeling studies [20, 71]. Therefore, we will omit the superscript *αβ* in the rest of steps.
2. The in-degree *K*_*i*,in_ and out-degree *K*_*i*,out_ for each neuron are generated by randomly sampling from the heterogeneous degree distributions that will be described later.
3. We use an iterative process to construct a binary adjacency matrix **A** = (*a*_*ij*_), where *a*_*ij*_ = 1, if there is a connection from neuron *j* to neuron *i*, or *a*_*ij*_ = 0 otherwise. Later we will use **A** = (*a*_*ij*_) to construct a weighted adjacency matrix. The connection probability between neurons *i* and *j* is proportional to the common neighbor factor, Γ_*ij*_. Initially we set Γ_*ij*_ = 1, but for subsequent iterations Γ_*ij*_ is a linear function of the number of common pre-synaptic neighbors *b*_*ij*_ shared by neuron *i* and neuron *j* is, as found in [3],
  $$\begin{array}{c} \Gamma_{ij}=1+\frac{b_{ij}-{\text{min}}_{i,j}\left(b_{ij}\right)}{{\text{max}}_{i,j}\left(b_{ij}\right)-{\text{min}}_{i,j}\left(b_{ij}\right)}\left(a_{\Gamma }-1\right), \end{array}$$ (1)
  where *b*_*ij*_ = [**A** **A**^*T*^]_*ij*_. The functions min_*i*,*j*_(⋅) and max_*i*,*j*_(⋅) denote the minimal and maximal values among all neuron pairs, respectively; *a*_Γ_ is the common neighbor coefficient, which is used to control the strength of the common neighbor effect. For instance, when *a*_Γ_ = 2 (the default value in our model), Eq 1 means that the common neighbor factor Γ_*ij*_ of the neuron pair with the most common neighbors is twice that of the pair with the least, and a linear interpolation in between.
4. The in-degree availability ${\tilde{K}}_{i,\text{in}}$ (denoting the number connections yet to be made) of all neurons are initialized to be their in-degrees, i.e. ${\tilde{K}}_{i,\text{in}}=K_{i,\text{in}}$. A neuron *j* is randomly selected from all neurons, and *K*_*j*,out_ connections from this neuron to other neurons are generated. When generating these connections, we need to ensure that the connection probability between any pair of neurons is proportional to both their distance-dependent factor Ω_*ij*_ and common neighbor factor Γ_*ij*_ as specified above, and that individual neurons have the specified in-degrees and out-degrees. To achieve this, we introduce a stochastic cost function $C_{ij}$ between neuron *j* and candidate post-synaptic neuron *i*,
  $$\begin{array}{c} C_{ij}=\frac{u_{ij}}{{\tilde{K}}_{i,\text{in}}\Omega_{ij}\Gamma_{ij}}, \end{array}$$ (2)
  where *i* = 1, 2, ⋯, *N*^*E*^ and *u*_*ij*_ is a random variable uniformly distributed between 0 and 1 capturing certain randomness in neural connectivity. Establish connections from neuron *j* to those post-synaptic neurons with the *K*_*j*,out_ smallest values of $C_{ij}$. For each connection made, the in-degree availability ${\tilde{K}}_{i,\text{in}}$ is reduced by one accordingly. Repeat the above procedures in this step for subsequently randomly chosen neuron *j*’s until the whole adjacency matrix **A**_*ij*_ is constructed.
5. Step 3 to Step 4 are repeated until the mean clustering coefficient (CC) of the network converges to a fixed value, which provides a single measure of the overall structural effect of the common neighbor factor as shown in the Results section (Fig 1*D*).

The second procedure of the algorithm is to set the synaptic efficacies (connection strengths) *J*_*ij*_ for the *a*_*ij*_ = 1 connections. Experimentally, the distribution of excitatory synaptic connection strengths has been found to be heavily-tailed in cortical neurons [6, 72] and can be fitted by a log-normal function as measured from rat visual cortex layer 5 [5]. We use a similar log-normal distribution of *J*_*ij*_, which has a mean of 4.0 nS and a standard deviation of 1.9 nS. In our spiking neuron model that will be described later, unitary excitatory post-synaptic potential (EPSP) and current (EPSC) magnitudes from the resting potential are proportional to the connection strength *J*_*ij*_. As a result, the majority (50%) of unitary EPSP magnitudes are less than 2 mV and a few (2%) are greater than 5 mV. The distribution of *J*_*ij*_ is truncated at 38.3 nS since values larger than this produce unrealistic unitary EPSP magnitudes above 20 mV.

It has been found that unlike the connection probability, connection strength does not strongly depend on the inter-neuron distance [4]. As a first-order approximation, we can assume connection strength is independent of distance. Nevertheless, naively randomly assigning the strength values would result in a situation where the average incoming connection strength 〈*J*_*ij*_〉_*j*_ of each neuron *i* is a linear function of its in-degree *K*_*i*,in_, that is, 〈*J*_*ij*_〉_*j*_ ∝ *K*_*i*,in_, where 〈⋅〉_*j*_ denotes an average over *j*. However, experimental studies have found an inverse square root scaling between average connection strength and in-degree, ${\langle J_{ij}\rangle }_{j}\propto 1/\sqrt{K_{i,\text{in}}}$ [26]. To incorporate this inverse square root scaling into our model, we develop a method called reverse pooling (see S2 Appendix for details of the method). Briefly, the method draws connection strength values for each neuron without replacement from a pool of values pre-sampled from a given distribution, which is ordered and then randomly separated into two sub-pools for each neuron. Because the two sub-pools have different mean values, the desired average incoming connection strength for each neuron can be achieved by randomly drawing pre-calculated numbers of values from each sub-pool.

#### Heterogeneous degree distributions

We now specify the heterogeneous degree distributions used in Step 2 of our generative connectivity model. Direct evidence of heterogeneous degree distributions of neural connectivity at the synaptic level is lacking, mainly due to small sample sizes as limited by currently available tracing techniques [25]. However, heavily-tailed degree distributions have been found in the effective connectivity of rodent somatosensory cortex using transfer entropy-based methods [7], which produce qualitatively similar results to those found by the patch clamp studies of [3]. Such degree distributions have also been observed in a biologically constrained *in silico* microcircuit of the same area [10]. Anatomically based estimates of the connectivity of layer 4 rat barrel cortex [9] have also indicated that the degree distributions of cortical neurons are significantly broader than Poisson distributions in the conventional E-R random networks (where the connection probabilities between any pair of neurons are identical) for a given mean value. In light of these studies, in our model, we assume that both the in- and out-degrees are heterogeneous, with some neurons having considerably more incoming and/or outgoing connections than expected in the E-R networks. More specifically, in our model, the in-degree *K*_in_ and out-degree *K*_out_ for any excitatory neuron are sampled from a hybrid distribution composed of a linear combination of a Poisson distribution and a log-normal distribution, which is a mathematically simple and well-known heavy-tailed distribution,

$$\begin{array}{cc} K_{\text{in}} & =\left(1-q\right)K_{\text{in}}^{P}+qK_{\text{in}}^{L},\\ K_{\text{out}} & =\left(1-q\right)K_{\text{out}}^{P}+qK_{\text{out}}^{L}, \end{array}$$ (3)

where *K*_in_ and *K*_out_ are rounded to the nearest integers, *q* is the hybrid parameter, $K_{\text{in}}^{P}$ and $K_{\text{out}}^{P}$ are drawn from Poisson distributions with mean *ζ* = *p*^*EE*^*N*^*E*^, *p*^*EE*^ = 0.16 is the overall E-to-E connection probability; $K_{\text{in}}^{L}$ and $K_{\text{out}}^{L}$ are drawn from log-normal distributions with the same mean *ζ* and a standard deviation that is 20% of the mean. Furthermore, to allow for a control of the correlation between in- and out-degree as a system parameter, the two Poisson random variables and the two log-normal random variables are generated with identical correlation coefficients of *ρ*_*K*_; in our model, we assume there is a weak positive correlation *ρ*_*K*_ = 0.13; note that positively correlated in- and out-degrees have been found in microscopic connectivity data [25]. In our model, the hybrid parameter *q* allows the interpolation between Poisson distributions as in E-R networks (*q* = 0) and log-normal distributions (*q* = 1); with the default hybrid parameter *q* = 0.4, the hybrid in- and out-degree distributions have a mean and standard deviation of 635 and 83, respectively. The total sums of in- and out-degrees in a network must be exactly equal, which is not guaranteed by sampling them from distributions with the same means; to satisfy this constraint, a random subset of sampled in- or out-degrees are adjusted by one so that any mismatch in the total sums is compensated for.

#### Equalized I-E ratios

In our model, we consider an essential neurophysiological feature of local cortical circuits, that is, the excitatory post-synaptic currents are proportional to the inhibitory ones, with a homogeneous ratio across all excitatory neurons, as found in mouse primary visual cortex layer 2/3 [18]. To model this in our heterogeneous circuit, we consider the I-E ratio $\zeta_{i}=\sum_{k=1}^{K_{i,\text{in}}^{EI}}J_{ik}^{EI}/\sum_{j}J_{ij}^{EE}$, where $K_{i,\text{in}}^{EI}$ denotes the number of connections (in-degree) received by excitatory neuron *i* from the inhibitory population and the connection strength values $J_{ij}^{EE}$ are determined by the reverse pooling method mentioned above. To equalize the I-E ratio *ζ*_*i*_ across the neurons to a desired network-wide ratio, that is, 〈*ζ*_*i*_〉 = *ζ*, the $J_{ik}^{EI}$ values for neuron *i* are sampled from a Gaussian distribution with a mean equal to $\zeta \sum_{j}J_{ij}^{EE}/K_{i,\text{in}}^{EI}$ and a standard deviation that is 25% of the mean. The I-E ratio *ζ* will be varied as a system parameter to explore the spatiotemporal dynamics of our cortical circuit model.

We now summarize the parameters used for generating the entire network. For most of the results, we use *N*^*E*^ = 63 × 63 = 3969 excitatory neurons and *N*^*I*^ = 1000 inhibitory neurons. The ratio between *N*^*E*^ and *N*^*I*^ is around 4, consistent with published measurements from rat visual cortex [73]. We consider the distance between two neighboring excitatory neurons (one grid unit) in our model to be 7.4 μm based on the measurements from rat primary visual cortex layer 5 [74]. Therefore, our network model contains approximately the same number of pyramidal neurons as a 0.22 mm^2^ square patch of cortical layer 5. Our results are not sensitive to the size of the network; we have obtained similar results for larger networks with sizes such as *N*^*E*^ = 103 × 103 = 10609 and *N*^*I*^ = 2673. For the exponential distance-dependent connection probability functions used in Step 1 of our generative connectivity model, the decay constants for the excitatory connections are $\tau_{D}^{EE}=8$ grid units and $\tau_{D}^{IE}=10$ grid units (corresponding to roughly 74 μm), which are consistent with experimentally measured ranges [3]. For the inhibitory connection ranges, we use $\tau_{D}^{II}=\tau_{D}^{EI}=20$ grid units. For simplicity, the in-degree distributions for the connections from and to the inhibitory population are Poisson with overall connection probabilities *p*^*EI*^ = *p*^*IE*^ = 0.2 and *p*^*II*^ = 0.4, taking into account the fact that the inhibitory connections are locally denser than excitatory ones [75, 76]. The rest of the connection strengths are assumed to be homogeneous and we use $J_{ij}^{IE}=J^{IE}=5\;\text{nS}$ and $J_{ik}^{II}=J^{II}=25\;\text{nS}$. Our connectivity model can be regarded as a generic local circuit model of mammalian cortex. However, considering the variations across species and cortical areas in general, e.g. [77], our model is most applicable to the layer 5 of rodent primary sensory cortices, whose connectivity properties are used to constrain the majority of the connectivity features in our model.

### A local cortical circuit model

Our generative connectivity model synthesizes distance dependence of connections with heterogeneous connectivity properties, such as heterogeneities in in- and out-degrees and in connection strengths, thus giving rise to a novel spatially extended, heterogeneous network. Based on this network structure, we then construct a spiking circuit model with conductance-based, leaky integrate-and-fire (LIF) neurons. The subthreshold membrane potential $V_{i}^{\alpha }$ of neuron *i* from population *α* evolves according to

$$\begin{array}{c} C\frac{dV_{i}^{\alpha }\left(t\right)}{dt}=-g_{L}\left[V_{i}^{\alpha }\left(t\right)-V_{L}\right]+I_{i,K}^{\alpha }\left(t\right)+I_{i,\text{rec}}^{\alpha }\left(t\right)+I_{i,\text{ext}}^{\alpha }\left(t\right), \end{array}$$ (4)

where *C* = 0.25 nF is the capacitance, *g*_*L*_ = 16.7 nS is the leaky conductance, *V*_*L*_ = −70 mV is the reversal potential for the leak current [78], $I_{i,K}^{\alpha }\left(t\right)$ is the potassium current that introduces spike frequency adaptation to the neurons [79], $I_{i,\text{rec}}^{\alpha }$ is the recurrent synaptic current received by neuron *i*, and $I_{i,\text{ext}}^{\alpha }$ is the external current. When the membrane potential reaches the threshold *V*_*th*_ = −50 mV, a spike is emitted and the membrane potential is reset to the potential *V*_*rt*_ = −60 mV for an absolute refractory period *τ*_*ref*_ = 4 ms [78]. The potassium current is given by $I_{i,K}^{\alpha }=-g_{i,K}^{\alpha }\left(t\right)\left(V_{i}^{\alpha }-V_{K}\right)$, where $g_{i,K}^{\alpha }\left(t\right)$ is the active potassium conductance and *V*_*K*_ = −85 mV [80]. The dynamics of the potassium conductance are described by

$$\begin{array}{c} \frac{dg_{i,K}^{\alpha }\left(t\right)}{dt}=-\frac{g_{i,K}^{\alpha }\left(t\right)}{\tau^{K}}+\Delta g_{K}\sum_{k}\delta \left(t-t_{i,k}^{\alpha }\right), \end{array}$$ (5)

where $t_{i,k}^{\alpha }$ is the time of the *k*^*th*^ spike emitted by neuron *i* from population *α*, Δ*g*_*K*_ = 10 nS and *τ*^*K*^ = 80 ms [80]. Because spike frequency adaptation has been primarily observed in cortical pyramidal neurons [81, 82], we only include such adaptation for excitatory neurons in our model.

The recurrent synaptic current $I_{i,\text{rec}}^{\alpha }\left(t\right)$ in Eq 4 is

$$\begin{array}{c} I_{i,\text{rec}}^{\alpha }\left(t\right)=\sum_{\beta }I_{i,\text{rec}}^{\alpha \beta }\left(t\right)=\sum_{\beta }\left[-g_{i}^{\alpha \beta }\left(t\right)\left(V_{i}^{\alpha }-V_{\text{rev}}^{\beta }\right)\right], \end{array}$$ (6)

where $g_{i}^{\alpha \beta }$ is the conductance of the recurrent current $I_{i,\text{rec}}^{\alpha \beta }$ from the pre-synaptic population *β*. The excitatory and inhibitory reversal potentials are $V_{\text{rev}}^{E}=0\;\text{mV}$ and $V_{\text{rev}}^{I}=-80\;\text{mV}$, respectively [78]. The conductance $g_{i}^{\alpha \beta }\left(t\right)$ is given by

$$\begin{array}{c} g_{i}^{\alpha \beta }\left(t\right)=\sum_{j=1}^{N^{\beta }}a_{ij}^{\alpha \beta }J_{ij}^{\alpha \beta }s_{ij}^{\alpha \beta }\left(t\right), \end{array}$$ (7)

where both the connection topology $a_{ij}^{\alpha \beta }$ and the strength $J_{ij}^{\alpha \beta }$ are specified by our generative connectivity model described above. The non-dimensional gating variable $s_{ij}^{\alpha \beta }\left(t\right)$ in Eq 7 describes the synaptic dynamics [83],

$$\begin{array}{c} \frac{ds_{ij}^{\alpha \beta }}{dt}=-\frac{s_{ij}^{\alpha \beta }}{\tau_{d}^{\beta }}+\sum_{k}h^{\beta }\left(t-t_{j,k}^{\beta }-d_{ij}^{\alpha \beta }\right)\left(1-s_{ij}^{\alpha \beta }\right), \end{array}$$ (8)

where $\tau_{d}^{\beta }$ is the conductance decay time constant, $t_{j,k}^{\beta }$ is the time of the *k*^*th*^ spike emitted by neuron *j* from population *β*, and $d_{ij}^{\alpha \beta }$ is the conduction delay drawn from a uniform random distribution between 0 and 4 ms. The term *h*^*β*^(*t*) is described by a rectangular pulse with unitary area, that is, $h^{\beta }\left(t\right)=1/\tau_{r}^{\beta }$, if $0\leq t\leq \tau_{r}^{\beta }$, and otherwise *h*^*β*^(*t*) = 0, where $\tau_{r}^{\beta }=1\;\text{ms}$. The $\left(1-s_{ij}^{\alpha \beta }\right)$ term in Eq 8 introduces the saturation effect, which is a physiologically realistic feature that ensures that the gating variable (fraction of open channels) cannot exceed 100% [84]. In our model, the time scale of the excitatory conductance is an approximation of both fast AMPA receptor mediated conductance and slow NMDA receptor mediated conductance; therefore, the excitatory synaptic decay time constant $\tau_{d}^{E}=5\;\text{ms}$ is larger than the inhibitory one $\tau_{d}^{I}=3\;\text{ms}$.

In our local cortical circuit model, the external currents are excitatory $I_{i,\text{ext}}^{\alpha }=J_{\text{ext}}s_{i,\text{ext}}^{\alpha }\left(V_{i}^{\alpha }-V_{\text{rev}}^{E}\right)$, where *J*_ext_ = 2 nS, each gating variable $s_{i,\text{ext}}^{\alpha }$ is driven by a Poisson spike train $t_{i,\text{ext}}^{\alpha }$ with a rate $\zeta_{\text{ext}}^{\alpha }$ and the saturation effect is ignored, $ds_{i,\text{ext}}^{\alpha }/dt=-s_{i,\text{ext}}^{\alpha }/\tau_{d}^{E}+h^{E}\left(t-t_{i,\text{ext}}^{\alpha }\right)$. We use $\zeta_{\text{ext}}^{I}=1000\;\text{Hz}$ and calibrate $\zeta_{\text{ext}}^{E}$ at 850 Hz, so that the average spontaneous firing rate of excitatory neurons is 3.0 ± 0.2 in a dynamic working regime of the model, within which a range of key *in vivo* findings can be captured; this working regime will be illustrated in the Results section. To provide a high-level description of our local cortical circuit model, we adapt the table templates in [85] to summarize the structural setup (see Table 1) and the neuron model parameters in our model (see Table 2).

**Table 1:** Summary of model definition.

Populations	Excitatory (E) and inhibitory (I)
Topology	2D square Cartesian grid with periodic boundaries
	Excitatory neurons on integer (idealized anatomical) coordinates
	Inhibitory neurons uniformly randomly positioned
Connectivity	Algorithmically generated and constrained by empirical data
E-to-E	In- and out-degree sampled from hybrid Poisson-lognormal distributions, and weakly correlated
	Connection probability between neuron pairs proportional to both a distance dependent factor and a common neighbor factor
	Connection strengths sampled from a lognormal distribution, with the total in-coming connection strength following an inverse square-root scaling w.r.t. the in-degree
I-to-E	In-degree sampled from a Poisson distribution
	Connection probability proportional to a distance dependent factor
	Connection strength sampled from a Gaussian distribution for each excitatory neuron with a given mean that equalizes the ratios between total incoming E and I connection strengths across excitatory neurons (equalized I-E ratio)
E-to-I	In-degree sampled from a Poisson distribution
	Connection probability proportional to a distance dependent factor
	Identical connection strength
I-to-I	Similar to E-to-I
Neuron model	Leaky integrate-and-fire neurons (Eq 4)
	Excitatory neurons with spike-frequency adaptation (Eq 5)
Channel model	Conductance-based excitatory and inhibitory channels (Eqs 6 and 7)
Synapse model	Fraction of open channels (gating variable) described by first-order dynamics driven by pulse inputs (incoming spikes; Eq 8)
External input	Individual neurons receive excitatory synaptic inputs driven by independent Poisson spike trains with constant rate

**Table 2:** Summary of neuron parameters (in a consistent system of units; see Eq 4).

Definition	Symbol	Value	Units
Capacitance	*C*	0.25	nF
Leaky conductance	*g*_*L*_	0.0167	*μ*S
Unit increase in potassium conductance	Δ*g*_*K*_	0.010	*μ*S
Reversal potential for leak current	*V*_*L*_	-70	mV
Reversal potential for postsynaptic currents	$V_{rev}^{E,I}$	0,-80	mV
Reversal potential for potassium currents	*V*_*K*_	-85	mV
Firing threshold	*V*_*th*_	-50	mV
Reset potential	*V*_*rt*_	-60	mV
Decay time constant for potassium conductance	*τ*^*K*^	80	ms
Rise time constant for postsynaptic conductance	$\tau_{r}^{E,I}$	1	ms
Decay time constant for postsynaptic conductance	$\tau_{d}^{E,I}$	5,3	ms
Absolute refractory period	*τ*_*ref*_	4	ms
Rate of external Poisson spike train	$\zeta_{\text{ext}}^{E,I}$	0.85, 1.0	kHz

#### Variance decomposition analysis

To study how different heterogeneities in the neural circuit model each impact neural dynamics, we develop the following variance decomposition analysis. As described in Eqs (6 and 7), the recurrent synaptic current in the conductance-based model is given by

$$\begin{array}{c} I_{i,\text{rec}}^{\alpha \beta }\left(t\right)=-\left[V_{i}^{\alpha }\left(t\right)-V_{\text{rev}}^{\beta }\right]\sum_{j=1}^{N^{\beta }}a_{ij}^{\alpha \beta }J_{ij}^{\alpha \beta }s_{j}^{\beta }\left(t\right). \end{array}$$ (9)

We obtain the deviation parts of the connection topology (denoted by $\delta a_{ij}^{\alpha \beta }$) and strength (denoted by $\delta J_{ij}^{\alpha \beta }$) by removing the mean values (over different realizations), that is, $\delta a_{ij}^{\alpha \beta }=a_{ij}^{\alpha \beta }-{\bar{a}}_{ij}^{\alpha \beta }$ and $\delta J_{ij}^{\alpha \beta }=J_{ij}^{\alpha \beta }-{\bar{J}}^{\alpha \beta }$. We denote the standard deviations as $\sigma_{ij,a}^{\alpha \beta }$ and $\sigma_{ij,J}^{\alpha \beta }$, respectively. Similarly, assuming the dynamics are stationary in terms of firing rate of each neuron, we have the time varying parts of the gating variable and the membrane potential as $\delta s_{j}^{\beta }\left(t\right)=s_{j}^{\beta }\left(t\right)-{\bar{s}}_{j}^{\beta }$ and $\delta V_{i}^{\alpha }\left(t\right)=V_{i}^{\alpha }\left(t\right)-{\bar{V}}_{i}^{\alpha }$, where the averages are taken over an ensemble of networks with different realizations of the random initial conditions. Following the same notation, the corresponding standard deviations are $\sigma_{j,s}^{\beta }$ and $\sigma_{i,V}^{\alpha }$.

Substituting the above expressions into Eq 9, expanding the brackets and after some derivations utilizing or assuming the independencies among the deviation and time varying parts (see S3 Appendix), we obtain the mean and time varying part of the recurrent current, $I_{i,\text{rec}}^{\alpha \beta }\left(t\right)={\bar{I}}_{i,\text{rec}}^{\alpha \beta }+\delta I_{i,\text{rec}}^{\alpha \beta }\left(t\right)$, where the mean is given by ${\bar{I}}_{i,\text{rec}}^{\alpha \beta }=-\left({\bar{V}}_{i}^{\alpha }-V_{\text{rev}}^{\beta }\right){\bar{J}}^{\alpha \beta }\sum_{j=1}^{N^{\beta }}{\bar{a}}_{ij}^{\alpha \beta }{\bar{s}}_{j}^{\beta }$ and the time varying part can be expressed as a sum of the products of 15 random variables and their associated standard deviations,

$$\begin{array}{c} \delta I_{i,\text{rec}}^{\alpha \beta }\left(t\right)=\sum_{X}{\hat{\sigma }}_{i,X}^{\alpha \beta }\xi_{i,X}^{\alpha \beta }\left(t\right), \end{array}$$ (10)

where $\xi_{i,X}^{\alpha \beta }\left(t\right)$ are independent normal random variables with zero mean and unit variance and the associated standard deviations ${\hat{\sigma }}_{i,X}^{\alpha \beta }$ can be systematically obtained and expressed in terms of the mean or (co-)variance of the four components in Eq 9, namely, $a_{ij}^{\alpha \beta },J_{ij}^{\alpha \beta },s_{j}^{\beta }\left(t\right)$ and $V_{i}^{\alpha }\left(t\right)$. The subscript *X* contains the shorthand letters of the components contributing to the term ${\hat{\sigma }}_{i,X}^{\alpha \beta }$ via their (co-)variances instead of mean values; for instance, for *X* = *as*, ${\hat{\sigma }}_{i,as}^{\alpha \beta }=\left|{\bar{V}}_{i}^{\alpha }-V_{\text{rev}}^{\beta }\right|{\bar{J}}^{\alpha \beta }\sqrt{\sum_{j}{\left(\sigma_{ij,a}^{\alpha \beta }\sigma_{j,s}^{\beta }\right)}^{2}}$ (see S3 Appendix for detailed derivations). And there are in total 15 different terms for the recurrent current from population *β* to *α*, that is, *X* ∈ {*a*, *J*, *s*, *V*, *aJ*, *as*, *aV*, *Js*, *JV*, *sV*, *JsV*, *asV*, *aJV*, *aJs*, *aJsV*}. These results enable us to obtain the variance *σ*^2^(⋅) of the synaptic currents,

$$\begin{array}{cc} \sigma^{2}\left[I_{i,\text{rec}}^{\alpha \beta }\left(t\right)\right]= & \sum_{X}{\left({\hat{\sigma }}_{i,X}^{\alpha \beta }\right)}^{2}, \end{array}$$ (11)

which allows us to decompose the contributions to the variance of recurrent currents from different sources, including connectivities.

### Statistical analysis

Simulations of the local cortical circuit model are performed using the forward Euler method with a time-step of 0.1 ms. The initial membrane potentials are uniformly distributed between *V*_rt_ = −60 mV and *V*_*th*_ = −50 mV. A typical trial is one realization of the circuit simulated for at least 100 biological seconds unless otherwise stated, with the first 500 ms excluded. All the standard errors of mean (SEM) reported in the Results section are calculated from 10 trials unless otherwise stated. Statistical analysis is done with MATLAB2015b. Spike train statistics are calculated with a bin size of 1 ms. The custom C++ simulation software and MATLAB codes used for analysis are available on GitHub (URL: https://github.com/BrainDynamicsUSYD/SpikeNet).

#### Localized propagating wave detection

The spontaneous activity of our local cortical circuit model exhibits rich spatiotemporal dynamics, including propagating wave patterns. To compare the properties of these wave patterns in our model with those reported in [14], we use the following detection method. We assume at any time the excitatory population has a firing rate profile of a 2-D circular Gaussian function, with the firing rate $v_{i}^{E}$ of excitatory neuron *i* described as

$$\begin{array}{c} v_{i}^{E}\left(\theta_{t}\right)=v_{p}\left(t\right)e^{-|r_{i}-r_{p}{\left(t\right)|}^{2}/2\sigma_{p}^{2}\left(t\right)}, \end{array}$$ (12)

where **r**_*i*_ is the coordinate vector of neuron *i* and $\theta_{t}=\left[r_{p}\left(t\right),\sigma_{p}^{2}\left(t\right),v_{p}\left(t\right)\right]$ is the parameter vector that contains the center position, the variance, and the height of the Gaussian function, respectively. We further assume that the firing process of each neuron around time *t* can be approximated by a Poisson process with rate $v_{i}^{E}\left(\theta_{t}\right)$. With this assumption, the log-likelihood ln *L*(***θ***_*t*_) of the parameters is given by

$$\begin{array}{c} \text{ln}\;L\left(\theta_{t}\right)=\sum_{i}[-v_{i}^{E}\left(\theta_{t}\right)+n_{i}^{E}\left(t\right)\;\text{ln}\;v_{i}^{E}\left(\theta_{t}\right)], \end{array}$$ (13)

where $n_{i}^{E}\left(t\right)$ is the spike count of excitatory neuron *i* within a small window Δ*t* = 5 ms centered around time *t*. To find the maximal likelihood fit to the spiking data on the grid with periodic boundaries, we transform the grid coordinates into points on unit circles and calculate the circular means. To measure the goodness of fit, we compare the Gaussian model against a null model that assumes a uniform firing rate profile by calculating the Bayes factor *B*_12_ (see S4 Appendix for details). A Bayes factor log_10_(*B*_12_) > 2 means the Gaussian model is significantly more strongly supported by the data than the null model is [86]. In our analysis, we regard any fitting results with log_10_(*B*_12_) < 2 as irregular firing states between coherent spiking wave patterns.

## Supporting information

S1 Appendix Diffusion-approximation-based analysis. (PDF)

S2 Appendix The reverse-pooling technique. (PDF)

S3 Appendix Variance decomposition analysis. (PDF)

S4 Appendix Bayes factor for the detected spiking wave. (PDF)

S1 Fig Rich-club connectivity and lognormal distribution of connection strengths emerge from transfer entropy-based effective connectivity. (PDF)

S1 Video Sample video for Fig 3*C*. The localized propagating wave state.
(MP4)

S2 Video Sample video for Fig 3*D*. The global propagating wave state.
(MP4)

S3 Video Sample video for Fig 3*E*. The transition state.
(MP4)

S4 Video Sample video for State II in Fig 6*E*. The video is visualized in the same way as in Fig 3*B*–3*E*. The parameters used for the critical case video are *ξ*/*ξ*_*c*_ = 1.15 and *a*_Γ_ = 2, where the excitatory population firing rate is 6.6 Hz.
(MP4)
