# Supplementary material for: Rich-club connectivity, diverse population coupling, and dynamical activity patterns emerging from local cortical circuits
Source: PLoS Comput Biol. 2019 Apr 2;15(4):e1006902. doi: 10.1371/journal.pcbi.1006902 (PMC6461296; doi:10.1371/journal.pcbi.1006902)
Supplement: S1 Appendix — (PDF) [file pcbi.1006902.s001.pdf]

S1 Appendix: Diffusion-approximation-based analysis

To understand the mechanism of how the stability of the spatially uniform, asynchronous state is lost as the I-E ratio varies in spatially-extended neural circuits, we adapt an analytical framework known as the diffusion approximation [1–3], in particular, its recent extension for analyzing spatially extended, spiking neural circuits [4]. This analysis preserves the following key biological features of the original model (Eq. 4): (a) conductance-based neurons; (b) separate excitatory and inhibitory populations; (c) distance-dependent probability for synaptic connections; (d) variances of connection strengths; and (e) fluctuations in the synaptic inputs. While keeping these biologically realistic features, a number of simplifications are made for analytical tractability, including instantaneous synaptic dynamics without rise or decay time, the removal of refractory period, synaptic saturation, and adaptation, and the removal of common neighbor effect as well as broadly distributed in- and out-degrees. The parameters used in this analysis are the same as those in our circuit model, except that the synaptic weights are rescaled appropriately to compensate the removal of synaptic time constants.

The core idea of the diffusion approximation is to replace the synaptic current driven by random input spikes with a Gaussian white noise of the same mean and variance, provided that the total input firing rate is sufficiently high and the contribution from individual spikes is small. Although the diffusion approximation is conventionally applied to analyzing randomly connected networks, it has been recently extended to the case of spatially structured networks [4]. The analysis involves two main steps. First, by solving a time-independent Fokker-Planck equation (FPE) for the membrane potential, the uniform steady state of the firing rate can be determined as a function of the input rates, that is, $r_0 = \phi(R_0^e, R_0^i)$, with R_0^e (R_0^i) being the total excitatory (inhibitory) input rate. Second, we analyze the linear stability of the spatially uniform states by considering the time dependent FPE under perturbations of the form, $\delta r = e^{\lambda t + i\mathbf{k}\cdot\mathbf{x}}$, and obtain the dispersion relation between the temporal eigenvalue λ and the 2D spatial wave vector $\mathbf{k}$. Therefore, the transition from the asynchronous state ($\mathrm{Re}[\lambda] < 0$) to the spatially heterogeneous ($\mathrm{Re}[\lambda] > 0$) state can be determined.

Based on the simplifications as outlined in the main text, we have the following model to approximate our microcircuit model:

$$\frac{dV_i^\alpha}{dt} = -\frac{V_i^\alpha - V_L}{\tau_L} + I_{i,\mathrm{rec}}^\alpha(t) + I_{i,\mathrm{ext}}^\alpha(t), \tag{14}$$

where $\alpha \in \{E, I\}$ is an index for excitatory and inhibitory populations; $\tau_L = C/g_L$ ms is the same membrane time constant as in the original model. The recurrent synaptic current is,

$$I_{i,\mathrm{rec}}^\alpha(t) = -\sum_{\beta,j} \hat{J}_{ij}^{\alpha\beta}(V - V_{\mathrm{rev}}^\alpha) a_{ij}^{\alpha\beta} s_j^\beta(t), \tag{15}$$

where $\hat{J}_{ij}^{\alpha\beta}$ is a non-dimensional parameter describing synaptic strengths, with a mean value of $\hat{J}^{\alpha\beta}$ and a standard deviation of $\hat{J}^{\alpha\beta}\Delta_{\alpha\beta}$, and $s_j^\beta(t) = \sum_k \delta(t - t_\alpha^k)$ is the Poisson spike train of neuron j in population β, with firing rate $r_j^\beta(t)$; note that the spike train $s_j^\beta(t)$ here has a unit of s^{-1} and is different from the gating variable used in the main text. The conversion to the non-dimensional synaptic strength $\hat{J}_{ij}^{\alpha\beta}$ from the strength $J_{ij}^{\alpha\beta}$ used in the full model (in the unit of conductance) is $\hat{J}_{ij}^{\alpha\beta} = J_{ij}^{\alpha\beta} A^\beta / C$,

where

$$A^{\beta} = \frac{\tau_d^{\beta}\tau_r^{\beta}}{\tau_d^{\beta}+\tau_r^{\beta}} + \tau_d^{\beta}(\frac{\tau_d^{\beta}}{\tau_d^{\beta}+\tau_r^{\beta}})^2(1-e^{-1-\tau_r^{\beta}/\tau_d^{\beta}}), \tag{16}$$

which is the integral of the time course of the gating variable (Eq. 8) in response to a single incoming spike. The binary random variable $a_{ij}^{\alpha\beta} \in \{0,1\}$ in Eq. 15 represents the connectivity from neuron j in population β to neuron i in population α, with distance-dependent probability,

$$p_{ij}^{\alpha\beta} = K_{\alpha\beta} w_{\alpha\beta}(\|\mathbf{x}_i^{\alpha} - \mathbf{x}_j^{\beta}\|), \tag{17}$$

where $\mathbf{x}_i^{\alpha} \in [-\pi,\pi)^2$ is the position of neuron i from population α, $K_{\alpha\beta}$ is the average in-degree, i.e. the average number of synapses from population β to population α, equal to that in the microcircuit model, and $w_{\alpha\beta}(x)$ is the connectivity density satisfying the normalization, $\int w_{\alpha\beta}(\|\mathbf{x}\|)d\mathbf{x} = 1$,

$$w_{\alpha\beta}(x) = \frac{1}{2\pi d_{\alpha\beta}^2}\exp\left(-\frac{x}{d_{\alpha\beta}}\right). \tag{18}$$

The external synaptic current in Eq. 14 is,

$$I_{i,\mathrm{ext}}^{\alpha} = -\hat{J}_{\mathrm{ext}}(V - V_{\mathrm{rev}}^{E})s_{\mathrm{ext}}^{\alpha}(t), \tag{19}$$

where $s_{\mathrm{ext}}^{\alpha}(t)$ is the external Poisson spike train with rate $R_{\mathrm{ext}}^{\alpha}$.

For the conductance-based network model (Eq. 14-19), the effect of a delta spike on the membrane potential is not uniquely defined. Here, we follow the definition in [3] and impose the rule of ordinary calculus; therefore, for an input spike arriving at $t_{\alpha}^{k} = 0$, the jump in membrane potential is $\Delta V_i^{\alpha} = -(1-e^{-\hat{J}_{ij}^{\alpha\beta}})(V_i^{\alpha} - E_{\alpha})$.

When the total input rate to each neuron is high and $\hat{J}_{ij}^{\alpha\beta} \ll 1$ such that the jumps in the membrane potential due to individual spikes are small, we can apply the diffusion approximation as in [2], in which the synaptic current is replaced by a white Gaussian noise with mean and variance as follows,

$$\mathbb{E}[I_{i,\mathrm{rec}}^{\alpha}(t)] = -\sum_{\beta,j} b_{\alpha\beta}(V - E_{\beta})p_{ij}^{\alpha\beta} r_j^{\beta}(t), \tag{20}$$

$$\mathrm{Var}[I_{i,\mathrm{rec}}^{\alpha}(t)] = \sum_{\beta,j} b_{\alpha\beta}^2(1+\Delta_{\alpha\beta}^2)(V - E_{\beta})^2 p_{ij}^{\alpha\beta} r_j^{\beta}(t), \tag{21}$$

where $\mathbb{E}[\cdot]$ denotes the ensemble average, i.e. the average over all realizations of possible networks. Note we use the approximation, $\mathbb{E}[1-e^{-\hat{J}_{ij}^{\alpha\beta}}] \approx 1 - e^{-\hat{J}^{\alpha\beta}} \equiv b_{\alpha\beta}$, which is accurate to $\mathcal{O}(b_{\alpha\beta}^2)$, and $\mathbb{E}[(1-e^{-\hat{J}_{ij}^{\alpha\beta}})^2] \approx b_{\alpha\beta}^2\Delta_{\alpha\beta}^2$, which is accurate to $\mathcal{O}(b_{\alpha\beta}^3)$; it is assumed that the firing activity of neurons at different locations is uncorrelated.

Extending to a spatial continuum, we can write down the total input Poisson rate from population β to population α as,

$$R_{\mathrm{rec}}^{\alpha\beta}(\mathbf{x},t) = K_{\alpha\beta}[w_{\alpha\beta} \otimes r^{\beta}](\mathbf{x},t) = K_{\alpha\beta}\int w_{\alpha\beta}(\|\mathbf{x}-\mathbf{y}\|)r^{\beta}(\mathbf{y},t)d\mathbf{y}, \tag{22}$$

where the symbol $\otimes$ denotes spatial convolution, and $r^{\alpha}(\mathbf{x},t)$ is the time-dependent firing rate.

Therefore, we obtain the following stochastic differential equation (SDE),

$$\partial_t V^{\alpha}(\mathbf{x},t) = -F_{\alpha}(V^{\alpha},\mathbf{x},t) + \sqrt{2D_{\alpha}(V^{\alpha},\mathbf{x},t)}\xi_t(\mathbf{x}), \tag{23}$$

with spatially uncorrelated unit Gaussian white noise $\xi_t(\mathbf{x})$. The corresponding Fokker-Planck equation describing the distribution of the membrane potential is,

$$\partial_t P_\alpha(v,\mathbf{x},t) = \partial_v[F_\alpha(v,\mathbf{x},t)P_\alpha(v,\mathbf{x},t)] + \partial_v^2[D_\alpha(v,\mathbf{x},t)P_\alpha(v,\mathbf{x},t)], \tag{24}$$

with appropriate boundary conditions as in [3]. The drift and the diffusion terms are,

$$F_\alpha(v,\mathbf{x},t) = \frac{v-V_L}{\tau_L} + b_{\alpha E}(v-V_{\text{rev}}^E)R_{\alpha E}(\mathbf{x},t) \\ + b_{\alpha I}(v-V_{\text{rev}}^I)R_{\alpha I}(\mathbf{x},t) + b_{\alpha,\text{ext}}(v-V_{\text{rev}}^E)R_{\text{ext}}^\alpha, \tag{25}$$

$$D_\alpha(v,\mathbf{x},t) = \frac{1}{2}[b_{\alpha E}^2(1+\Delta_{\alpha E}^2)(v-V_{\text{rev}}^E)^2R_{\alpha E}(\mathbf{x},t) \\ + b_{\alpha I}^2(1+\Delta_{\alpha I}^2)(v-V_{\text{rev}}^I)^2R_{\alpha I}(\mathbf{x},t) + b_{\alpha,\text{ext}}^2(v-V_{\text{rev}}^E)^2R_{\text{ext}}^\alpha]. \tag{26}$$

The FPE allows us to extract useful information about how the spiking network generates spatially localized activity patterns, as we show below.

Stationary firing rate and stability

We then conduct a stability analysis on the spatially uniform firing rate derived from the stationary FPE, following the methods developed in [4]. The goal is to determine numerically the temporal eigenvalues of the firing rate with respect to spatially periodic perturbations. First, consider the spatially uniform steady state, $r^\alpha(\mathbf{x},t) = r_0^\alpha$. Under this condition, the firing rate is stationary, and by numerically solving the time-independent FPE (setting time derivative in Eq. 24 to zero), we can numerically obtain a transfer function $\phi(\cdot)$ that relates the stationary firing rate to the total stationary input rates [3, 5],

$$r_0^\alpha = \phi^\alpha[R_0^{\alpha e}, R_0^{\alpha i}]. \tag{27}$$

Substitute the uniform solution r_0^α to Eq. 22, we find that the total input rates are simplified to $R_0^{\alpha\beta} = K_{\alpha\beta}r_0^\beta$, and r_0^α thus can be determined by numerically solving Eq. 27.

Next, we look at how output firing rate changes in response to small changes in the input rates. Consider time-dependent input rate of the form,

$$R_{\text{rec}}^{\alpha\beta}(\mathbf{x},t) = R_0^{\alpha\beta} + R_1^{\alpha\beta}(\mathbf{x})e^{i\omega t}, \tag{28}$$

which describes weak periodic modulation around the uniform steady state. The corresponding firing rate of the neuron is then,

$$r^\alpha(\mathbf{x},t) = r_0^\alpha + r_1^\alpha(\mathbf{x};\omega)e^{i\omega t}. \tag{29}$$

By using the time-dependent FPE, we can numerically obtain the susceptibility $\tilde{\chi}^{\alpha\beta}(\omega) = r_1^\alpha/R_1^{\alpha\beta}$, a complex-valued variable whose amplitude describes the firing rate gain with respect to sinusoidal modulation in the input firing rate $R^{\alpha\beta}(t)$, and whose argument describes the phase shift [3, 5]. The inverse Fourier transform of the susceptibility gives the impulse response function in the temporal domain, $\chi^{\alpha\beta}(t) = \frac{1}{2\pi}\int_{-\infty}^{\infty}\tilde{\chi}^{\alpha\beta}(\omega)e^{i\omega t}d\omega$. The linear response to an arbitrary temporally-varying perturbation is simply the temporal convolution (denoted with '$*$') with the impulse response function; therefore, we have,

$$\delta r^\alpha(\mathbf{x},t) = \sum_\beta[\chi^{\alpha\beta}(t) * \delta R^{\alpha\beta}](\mathbf{x},t) = \sum_\beta K_{\alpha\beta}[\chi^{\alpha\beta}(t) * w_{\alpha\beta}\otimes\delta r^\alpha](\mathbf{x},t). \tag{30}$$

Substituting in the time-dependent ansatz, $r^{\alpha}(\mathbf{x}, t) = r_0^{\alpha} + \bar{r}_{\alpha} e^{\lambda t + i\mathbf{k}\cdot\mathbf{x}}$, which describes the first-order dynamics of a spatially periodic perturbation, we obtain,

$$\bar{r}_{\alpha} = \sum_{\beta} K_{\alpha\beta} \tilde{w}_{\alpha\beta}(\mathbf{k}) \hat{\chi}^{\alpha\beta}(\lambda) \bar{r}_{\beta}, \tag{31}$$

with the Fourier coefficient, $\tilde{w}_{\alpha\beta}(\mathbf{k}) = \int w_{\alpha\beta}(\|\mathbf{x}\|) e^{-i\mathbf{k}\cdot\mathbf{x}} d\mathbf{x}$, and the Laplace transform, $\hat{\chi}^{\alpha\beta}(\lambda) = \int_0^{\infty} \chi^{\alpha\beta}(t) e^{-\lambda t} dt$. This has non-trivial solution only if,

$$\det[M(\lambda; \mathbf{k}) - I] = 0, \tag{32}$$

where

$$M(\lambda; \mathbf{k}) = \begin{bmatrix} K_{EE}\hat{\chi}^{EE}(\lambda)\tilde{w}_{EE}(\mathbf{k}) & K_{EI}\hat{\chi}^{EI}(\lambda)\tilde{w}_{EI}(\mathbf{k}) \\ K_{IE}\hat{\chi}^{IE}(\lambda)\tilde{w}_{IE}(\mathbf{k}) & K_{II}\hat{\chi}^{II}(\lambda)\tilde{w}_{II}(\mathbf{k}) \end{bmatrix}. \tag{33}$$

Therefore, by numerically solving Eq. 32 for the temporal eigenvalues $\lambda \in \mathbb{C}$ for each $\mathbf{k} \in \mathbb{N}^2$, we can determine the stability of the spiking network through the sign of $\mathrm{Re}[\lambda]$.

References

1. Amit DJ, Brunel N. Model of global spontaneous activity and local structured activity during delay periods in the cerebral cortex. Cerebral Cortex. 1997;7(3):237–252.

2. Brunel N. Dynamics of sparsely connected networks of excitatory and inhibitory neurons. Journal of Computational Neuroscience. 2000;8:183–208. doi:10.1016/S0928-4257(00)01084-6.

3. Richardson MJE. Firing-rate response of linear and nonlinear integrate-and-fire neurons to modulated current-based and conductance-based synaptic drive. Physical Review E. 2007;76(2):1–15. doi:10.1103/PhysRevE.76.021919.

4. Pyle R, Rosenbaum R. Spatiotemporal dynamics and reliable computations in recurrent spiking neural networks. Physical Review Letters. 2017;118(1):018103.

5. Rosenbaum R. A Diffusion Approximation and Numerical Methods for Adaptive Neuron Models with Stochastic Inputs. Frontiers in Computational Neuroscience. 2016;10(April):1–20. doi:10.3389/fncom.2016.00039.
