# Supplementary material for: Rich-club connectivity, diverse population coupling, and dynamical activity patterns emerging from local cortical circuits
Source: PLoS Comput Biol. 2019 Apr 2;15(4):e1006902. doi: 10.1371/journal.pcbi.1006902 (PMC6461296; doi:10.1371/journal.pcbi.1006902)
Supplement: S2 Appendix — (PDF) [file pcbi.1006902.s002.pdf]

S2 Appendix: The reverse-pooling technique

As pointed out in the Materials and Methods section, randomly sampling the connection strength values from a given distribution and assigning them to the existing connections would result in the situation that the average incoming connection strength $\langle J_{ij}\rangle_j$ of each neuron i is a linear function of its in-degree $K_{i,\text{in}}$, that is, $\langle J_{ij}\rangle_j \propto K_{i,\text{in}}$. In order to obtain a more general relationship between $\langle J_{ij}\rangle_j$ and $K_{i,\text{in}}$ other than linear, we develop the following algorithm.

1. Generate a pool of connection strengths J_p by sampling from the given distribution with the exact pool size $\sum_i K_{i,\text{in}}$. These values J_p in pool will be assigned to the existing connections a_{ij}. The pool is sorted so that $J_p \leq J_{p+1}$. Denote the current pool size (the number of values left in the pool yet to be assigned) as P, which is $\sum_i K_{i,\text{in}}$ initially.

2. Randomly select a neuron i from the ones for which the connection strengths are not assigned yet.

3. In order to achieve a desired average incoming connection strength for neuron i, denoted by $\langle J_{ij}\rangle_j^*$, we randomly generate an integer $1 \leq s < P$ to divide the current pool into the left pool $1 \leq p \leq s$ and the right pool $s+1 < p \leq P$.

4. Check if the following equations have real solutions $N_L, N_R \geq 1$

$$N_L + N_R = K_{i,\text{in}}, \tag{34}$$

$$N_L \sum\nolimits_{p=1}^{s} J_p/s + N_R \sum\nolimits_{p=s+1}^{P} J_p/(P-s) = K_{i,\text{in}}\langle J_{ij}\rangle_j^*. \tag{35}$$

   If not, go back to Step 3. (This condition is relaxed when there are only a few neurons' connection strengths left to be assigned.)

5. Round N_L and N_R to the nearest integers and randomly select N_L and N_R values from the left pool and the right pool, respectively. Randomly assign them to a_{ij}, where $j = 1, 2, \ldots K_{i,\text{in}}^{EE}$, without replacement (i.e., removing these values from the pool) and update the current pool size P. This ensures that the given distribution of connection strength will always be satisfied. Intuitively, steps 3-4 randomly divide the pool into two sub-pools for each neuron i, one with smaller values and the other with larger values, and step 5 calculates the correct numbers of values to be drawn from the two sub-pools to achieve a desired average incoming connection strength for this neuron.

6. Repeat from Step 2 to Step 5 until all the neurons' connection strength values J_{ij} are "reverse-pooled" from the original pool of connection strengths J_p.

The above reverse-pooling technique allows a generic relationship between the average incoming connection strength and the in-degree of each neuron, e.g. the experimentally found inverse square root scaling $\langle J_{ij}\rangle_j^* \propto 1/\sqrt{K_{i,\text{in}}}$ [1], to be used.

References

1. Barral J, Reyes AD. Synaptic scaling rule preserves excitatory–inhibitory balance and salient neuronal network dynamics. Nature. 2016;201:6.
