# Supplementary material for: Rich-club connectivity, diverse population coupling, and dynamical activity patterns emerging from local cortical circuits
Source: PLoS Comput Biol. 2019 Apr 2;15(4):e1006902. doi: 10.1371/journal.pcbi.1006902 (PMC6461296; doi:10.1371/journal.pcbi.1006902)
Supplement: S3 Appendix — (PDF) [file pcbi.1006902.s003.pdf]

S3 Appendix: Variance decomposition analysis

Here we give detailed derivations of the key results of variance decomposition analysis. As mentioned in the Materials and Methods section, the deviation or time varying parts of the variables in the recurrent current dynamics Eq. 9 can be expressed as $\delta s_j^\beta(t) = s_j^\beta(t) - \bar{s}_j^\beta$, $\delta V_i^\alpha(t) = V_i^\alpha(t) - \bar{V}_i^\alpha$, $\delta a_{ij}^{\alpha\beta} = a_{ij}^{\alpha\beta} - \bar{a}_{ij}^{\alpha\beta}$ and $\delta J_{ij}^{\alpha\beta} = J_{ij}^{\alpha\beta} - \bar{J}^{\alpha\beta}$. Substituting them into Eq. 9 and expanding the brackets gives

$$\begin{aligned} I_{i,\mathrm{rec}}^{\alpha\beta}(t) = &-(\bar{V}_i^\alpha - V_{\mathrm{rev}}^\beta)\bar{J}^{\alpha\beta}\sum_{j=1}^{N^\beta}\bar{a}_{ij}^{\alpha\beta}\bar{s}_j^\beta \\ &-(\bar{V}_i^\alpha - V_{\mathrm{rev}}^\beta)\bar{J}^{\alpha\beta}\sum_{j=1}^{N^\beta}\bar{s}_j^\beta\delta a_{ij}^{\alpha\beta} \qquad (36) \\ &-(\bar{V}_i^\alpha - V_{\mathrm{rev}}^\beta)\bar{J}^{\alpha\beta}\sum_{j=1}^{N^\beta}\bar{a}_{ij}^{\alpha\beta}\delta s_j^\beta \qquad (37) \\ &-(\bar{V}_i^\alpha - V_{\mathrm{rev}}^\beta)\sum_{j=1}^{N^\beta}\bar{a}_{ij}^{\alpha\beta}\bar{s}_j^\beta\delta J_{ij}^{\alpha\beta} \\ &-\bar{J}^{\alpha\beta}\sum_{j=1}^{N^\beta}\bar{a}_{ij}^{\alpha\beta}\bar{s}_j^\beta\delta V_i^\alpha \\ &-12 \text{ remaining cross terms,} \end{aligned}$$

where $j = 1, 2, \cdots, N^\beta$. For any neuron i, by the construction of our model, the deviations of the connection topology $\delta a_{ij}^{\alpha\beta}$ and strength $\delta J_{ij}^{\alpha\beta}$ are mutually independent and have no covariances among themselves; also, $\delta a_{ij}^{\alpha\beta}$ and $\delta J_{ij}^{\alpha\beta}$ are independent from the dynamic variables, namely, the time varying parts of the pre-synaptic gating variables $\delta s_j^\beta(t)$ and the post-synaptic membrane potential $\delta V_i^\alpha(t)$. In general, $\delta s_j^\beta(t)$, where $j = 1, 2, \cdots, N^\beta$, are not independent from each other and therefore we consider the covariances among them. For simplicity, we assume that, for neuron i, $\delta V_i^\alpha(t)$ is independent from $\delta s_j^\beta(t)$. These independencies either by definition or assumption allow us to simplify the above expansion. Given the fact that $N^\beta \gg 1$ and further assuming that the Lindeberg's condition is satisfied, the Lindeberg-Feller Central Limit Theorem (CLT) can be applied to the summation Eq. 36, which yields

$$\begin{aligned} -(\bar{V}_i^\alpha - V_{\mathrm{rev}}^\beta)\bar{J}^{\alpha\beta}\sum_{j=1}^{N^\beta}\bar{s}_j^\beta\delta a_{ij}^{\alpha\beta} &= |\bar{V}_i^\alpha - V_{\mathrm{rev}}^\beta|\bar{J}^{\alpha\beta}\sqrt{\sum\nolimits_j(\bar{s}_j^\beta\sigma_{ij,a}^{\alpha\beta})^2}\xi_{i,a}^{\alpha\beta} \qquad (38) \\ &= \hat{\alpha}_{i,a}^{\alpha\beta}\xi_{i,a}^{\alpha\beta}, \end{aligned}$$

where ξ (regardless of the super- or sub-scripts) denotes a standard normal random variable, any negative sign is absorbed into ξ, and $\sigma_{ij,a}^{\alpha\beta}$ denotes the standard deviation of $\delta a_{ij}^{\alpha\beta}$.

Although the CLT cannot be applied to the summation Eq. 37, as an approximation,

a normal random variable should still well describe the first two moments,

$$
\begin{aligned}
-(\bar{V}_i^{\alpha} - V_{\text{rev}}^{\beta}) \sum_{j=1}^{N^{\beta}} \bar{a}_{ij}^{\alpha\beta} \delta s_j^{\beta}(t) &= |\bar{V}_i^{\alpha} - V_{\text{rev}}^{\beta}| \sqrt{\sum\nolimits_{jk} \text{cov}(\bar{a}_{ij}^{\alpha\beta} \delta s_j^{\beta}, \bar{a}_{ik}^{\alpha\beta} \delta s_k^{\beta})} \xi_{i,s}^{\alpha\beta} \\
&= |\bar{V}_i^{\alpha} - V_{\text{rev}}^{\beta}| \sqrt{\sum\nolimits_{jk} \bar{a}_{ij}^{\alpha\beta} \bar{a}_{ik}^{\alpha\beta} \text{cov}(\delta s_j^{\beta}, \delta s_k^{\beta})} \xi_{i,s}^{\alpha\beta} \\
&= \hat{\alpha}_{i,s}^{\alpha\beta} \xi_{i,s}^{\alpha\beta}.
\end{aligned}
\tag{39}
$$

The summations of the products of random variables in the cross terms can be expressed in terms of normal random variables in the similar fashion, for example,

$$
\begin{aligned}
-(\bar{V}_i^{\alpha} - V_{\text{rev}}^{\beta}) \sum\nolimits_j \delta a_{ij}^{\alpha\beta} \delta s_j^{\beta}(t) &= |\bar{V}_i^{\alpha} - V_{\text{rev}}^{\beta}| \sqrt{\sum\nolimits_{jk} \text{cov}(\delta a_{ij}^{\alpha\beta} \delta s_j^{\beta}, \delta a_{ik}^{\alpha\beta} \delta s_k^{\beta})} \xi_{i,as}^{\alpha\beta} \\
&= |\bar{V}_i^{\alpha} - V_{\text{rev}}^{\beta}| \sqrt{\sum\nolimits_{jk} \text{cov}(\delta a_{ij}^{\alpha\beta}, \delta a_{ik}^{\alpha\beta}) \text{cov}(\delta s_j^{\beta}, \delta s_k^{\beta})} \xi_{i,as}^{\alpha\beta} \\
&= |\bar{V}_i^{\alpha} - V_{\text{rev}}^{\beta}| \sqrt{\sum\nolimits_{j=k} \text{cov}(\delta a_{ij}^{\alpha\beta}, \delta a_{ik}^{\alpha\beta}) \text{cov}(\delta s_j^{\beta}, \delta s_k^{\beta})} \xi_{i,as}^{\alpha\beta} \\
&= |\bar{V}_i^{\alpha} - V_{\text{rev}}^{\beta}| \sqrt{\sum\nolimits_j (\sigma_{ij,a}^{\alpha\beta} \sigma_{j,s}^{\beta})^2} \xi_{i,as}^{\alpha\beta}. \\
&= \hat{\alpha}_{i,as}^{\alpha\beta} \xi_{i,as}^{\alpha\beta}
\end{aligned}
\tag{40}
$$

In summary, the standard deviations $\hat{\alpha}_{i,X}^{\alpha\beta}$ in Eq. 10 , the key result of the variance decomposition analysis, can be systematically obtained in the above fashion. Note that the only terms where the covariance of $\delta s_j^{\beta}(t)$ contributes are $X \in \{s, sV\}$.

References

1. Schwarz G. Estimating the dimension of a model. The Annals of Statistics. 1978;6(2):461–464.

2. Kass RE, Raftery AE. Bayes factors. Journal of the American Statistical Association. 1995;90(430):773–795.
