# Supplementary material for: Rich-club connectivity, diverse population coupling, and dynamical activity patterns emerging from local cortical circuits
Source: PLoS Comput Biol. 2019 Apr 2;15(4):e1006902. doi: 10.1371/journal.pcbi.1006902 (PMC6461296; doi:10.1371/journal.pcbi.1006902)
Supplement: S4 Appendix — (PDF) [file pcbi.1006902.s004.pdf]

S4 Appendix: Bayes factor for the detected spiking wave

To measure the goodness of fit of the Gaussian firing rate profile model of the wave pattern to the spiking activity data, as mentioned in the Materials and Methods section, we calculate the Bayes factor of the Gaussian model against a null model that assumes a uniform firing rate profile. The Bayes factor, denoted by B_{12}, is defined as the ratio of the probabilities of the spiking activity data given by the two models and can be calculated by using the Schwarz approximation [1],

$$\ln B_{12} \approx \ln \hat{L}_1 - \ln \hat{L}_2 - \frac{1}{2}(k_1 - k_2) \ln N_s, \tag{41}$$

where $\hat{L}_1$ and $\hat{L}_2$ are the maximum likelihood values of the two models in comparison, N_s is the sample size and k_1 and k_2 are the number of model parameters. This approximation should provide a reasonable indication for the model comparison in large samples ($N \gg k_1, k_2$) [2]. In our case $k_1 = 4, k_2 = 1$ while N_s is the number of spikes binned within a time window of width $\Delta t = 5$ ms, which is around 60 given the average firing rate (3 Hz) and population size ($N^E = 3969$).

References

1. Schwarz G. Estimating the dimension of a model. The Annals of Statistics. 1978;6(2):461–464.
2. Kass RE, Raftery AE. Bayes factors. Journal of the American Statistical Association. 1995;90(430):773–795.
