# Supplementary material for: Rich-club connectivity, diverse population coupling, and dynamical activity patterns emerging from local cortical circuits
Source: PLoS Comput Biol. 2019 Apr 2;15(4):e1006902. doi: 10.1371/journal.pcbi.1006902 (PMC6461296; doi:10.1371/journal.pcbi.1006902)
Supplement: S1 Fig — (PDF) [file pcbi.1006902.s005.pdf]

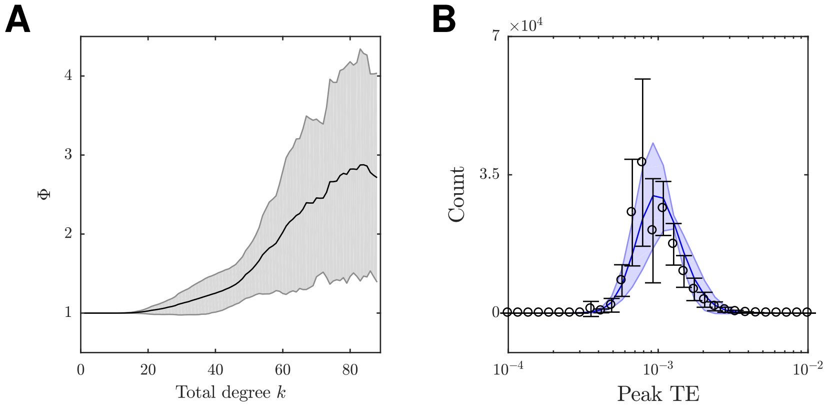

Fig S1. Rich-club connectivity and lognormal distribution of connection strengths emerge from transfer entropy based effective connectivity. (*A*) Normalized rich-club coefficients $\Phi(k)$ versus the total degree k, calculated in the same way as in Fig 2*B* (blue) in the main text using a random sample of 500 excitatory neurons from a circular region in the network containing 2000 excitatory neurons for each trial. The solid curve shows the average of 5 trials and the shaded area shows one SEM. **(*B*)** The distribution of the effective connection strengths, measured by the peak transfer entropy (peak TE), with the log-normal fit (blue solid line). The error bars and shaded area show one SEM from 5 trials. To obtain the effective connectivity, we first calculate the first-order TE measured at delays from 1 to 30 ms time bins for each neuron pair as in [1]. The maximum height of the TE-delay curve gives the peak TE, which is used as the effective connection strength. To distinguish significant connections measured by peak TE from those produced by chance, we jitter the spikes from the source neuron in each neuron pair and calculate the average peak TE and the average coincidence index (CI, which measures the narrowness of the peak; see [1] for more details) over 100 realizations of such randomization as in [2]. We reject those effective connections with peak TE and CI values that can be largely explained away by the jittered data using the same methods detailed in [2] (with a rejection threshold of 0.15).

References

1. Ito S, Hansen ME, Heiland R, Lumsdaine A, Litke AM, Beggs JM. Extending transfer entropy improves identification of effective connectivity in a spiking cortical network model. PloS ONE. 2011;6(11):e27431.

2. Nigam S, Shimono M, Ito S, Yeh FC, Timme N, Myroshnychenko M, et al. Rich-club organization in effective connectivity among cortical neurons. Journal of Neuroscience. 2016;36(3):670–684.
